# Rational design of drug-like compounds targeting *Mycobacterium marinum* MelF protein

**DOI:** 10.1371/journal.pone.0183060

**Published:** 2017-09-05

**Authors:** Renu Dharra, Sakshi Talwar, Yogesh Singh, Rani Gupta, Jeffrey D. Cirillo, Amit K. Pandey, Mahesh Kulharia, Promod K. Mehta

**Affiliations:** 1 Centre for Biotechnology, Maharshi Dayanand University (MDU), Rohtak, India; 2 Translational Health Science and Technology Institute (THSTI), Faridabad, India; 3 Department of Microbiology, University of Delhi South Campus, New Delhi, India; 4 Department of Microbial and Molecular Pathogenesis, Texas A&M Health Science Center, College Station, Texas, United States of America; 5 School of Basic and Applied Science, Central University of Punjab, Bathinda, India; Institut de Pharmacologie et de Biologie Structurale, FRANCE

## Abstract

The mycobacterial *mel2* locus (mycobacterial enhanced infection locus, Rv1936-1941) is *Mycobacterium marinum* and *M*. *tuberculosis* specific, which can withstand reactive oxygen species (ROS) and reactive nitrogen species (RNS) induced stress. A library of over a million compounds was screened using *in silico* virtual ligand screening (VLS) to identify inhibitors against the modeled structure of MelF protein expressed by *melF* of *mel2* locus so that *M*. *marinum’*s ability to withstand ROS/RNS stress could be reduced. The top ranked 1000 compounds were further screened to identify 178 compounds to maximize the scaffold diversity by manually evaluating the interaction of each compound with the target site. *M*. *marinum melF* was cloned, expressed and purified as maltose binding protein (MBP)-tagged recombinant protein in *Escherichia coli*. After establishing the flavin dependent oxidoreductase activity of MelF (~ 84 kDa), the inhibitors were screened for the inhibition of enzyme activity of whole cell lysate (WCL) and the purified MelF. Amongst these, 16 compounds could significantly inhibit the enzyme activity of purified MelF. For the six best inhibitory compounds, the minimal inhibitory concentration (MIC) was determined to be 3.4–19.4 μM and 13.5–38.8 μM for *M*. *marinum* and *M*. *tuberculosis*, respectively. Similarly, the minimal bactericidal concentration (MBC) was determined to be 6.8–38.8 μM and 27–38.8 μM against *M*. *marinum* and *M*. *tuberculosis*, respectively. One compound each in combination with isoniazid (INH) also showed synergistic inhibitory effect against *M*. *marinum* and *M*. *tuberculosis* with no cytotoxicity in HeLa cells. Interestingly, these inhibitors did not display any non-specific protein-structure destabilizing effect. Such inhibitors targeting the anti-ROS/RNS machinery may facilitate the efficient killing of replicating and nonreplicating mycobacteria inside the host cells.

## Introduction

Tuberculosis (TB), the leading public health problem stands along with human immunodeficiency virus (HIV) as the top most killers worldwide among the infectious diseases [1]. In 2015, 10.4 million new TB cases were estimated worldwide, of which 1.2 million people (11%) were HIV-positive [2]. TB epidemic has been exacerbated further by the emergence of multi-, extensively- and totally-drug resistant strains and there is an urgent need to devise new drugs. The most widely used strategy to identify drug targets is the whole-cell screening (WCS), which has been successfully used for the identification of new drug candidates such as diarylquinolines (TMC207) that target ATP biosynthesis, and benzathiazines (BTZ043) that target cell-wall arabinan biosynthesis [3,4]. The major disadvantage of WCS approach is the lack of precise target knowledge, which may result in the selection of unspecific toxic compounds [5]. Moreover, the compounds might be optimized for targets required only under *in vitro* culture conditions but are dispensable *in vivo*. Genome-derived, target-based approach has also been widely used to identify new anti-TB drugs [6], but it does not ensure the druggability. Virtual ligand screening (VLS) has been established as an efficient approach for drug design with a 100–1000 fold higher enrichment rate in comparison to conventional chemical screening [7,8]. Though this strategy is robust, it still requires human input in the form of identification of a druggable pocket, diversification of interacting moieties and the use of homology modeled structures [7,9].

We identified a novel *M*. *marinum* and *M*. *tuberculosis* specific *mel2* locus, which can withstand enhanced ROS and RNS stress [10–12] and has been suggested to play a role in *M*. *tuberculosis* lipid biosynthesis [13]. Among six gene of *mel2* locus identified in *M*. *marinum* and *M*. *tuberculosis* by *in silico* analysis; *melF*, *melG and melH* are close homologs of *luxA*, *luxG* and *luxH* genes involved in bioluminescence [12]. The *mel2* locus confers resistance to ROS/RNS stress in laboratory media and has a similar role in activated macrophages [11,12]. The MelF protein is a putative flavin dependent oxidoreductase and the mutation in *melF* displays a polar effect on the downstream genes of *mel2* locus [11]. The increased susceptibility of *melF* mutant to ROS/RNS could be partially retrieved by *melF* alone and completely by the entire *mel2* locus [11]. The role of *mel2* locus in resistance to ROS has been demonstrated for the persistence and dissemination of *M*. *tuberculosis* in C57BL/6 mice [14]. The oxidative stress has also been shown to enhance the susceptibility of mycobacterial cells to existing anti-TB drugs such as INH, clofazamine, etc. [15,16]. However, the development of new drugs is hampered by the slow growth of *M*. *tuberculosis*. Alternatively, a fast-grower, *M*. *marinum* is considered as a surrogate model to study *M*. *tuberculosis* pathogenesis [10,17] and the feasibility of using *M*. *marinum* as an anti-TB activity evaluation model has also been described [17]. Therefore, a study was designed to identify putative inhibitors by VLS analysis targeting MelF that can diminish the ability of *M*. *marinum* to withstand ROS/RNS stress and the results were validated by evaluating the flavin oxidoreductase activity of MelF protein and the growth inhibition/killing of bacteria.

## Materials and methods

### Reagents and chemicals

Restriction enzymes and modifying enzymes were purchased from New England Biolabs, Inc. (Beverly, Mass.) and were used according to the manufacturer’s guidelines. Shimadzu Model UV-2450 was used for all the spectrophotometric analysis. The template structure was downloaded from RCSB PDB (Protein Data Bank) [18] site. The ChemBridge's CORELibrary and Express-Pick Collection Library used for virtual ligand screening (VLS) were downloaded from www.chembridge.com. 178 drug-like compounds were purchased from ChemBridge, San Diego, USA for *in vitro* screening.

### Bacterial strains and growth conditions

The departmental committee of THSTI, Faridabad has given approval to work on *M*. *marinum* strain M in biosafety-hood and *M*. *tuberculosis* H_37_Rv in biosafety level-III facility at the International Centre for Genetic Engineering and Biotechnology, New Delhi. *M*. *marinum* strain M and *M*. *tuberculosis* H_37_Rv were grown at 33°C for ~ 7 days and at 37°C for ~ 3 weeks, respectively in 7H9 Middlebrook medium supplemented with 0.5% glycerol, 10% albumin dextrose complex (ADC) and 0.05% Tween-80 (M-ADC-TW) as detailed earlier [10,14]. The mycobacterial colony-forming units (CFU) were determined on 7H10 (M-ADC) agar [10,14]. *E*. *coli* XL1 Blue and BL21 strains were grown in Luria-Bertani (LB) medium at 37°C.

### Homology modeling, validation and druggable pocket definition

**Fig 1:**
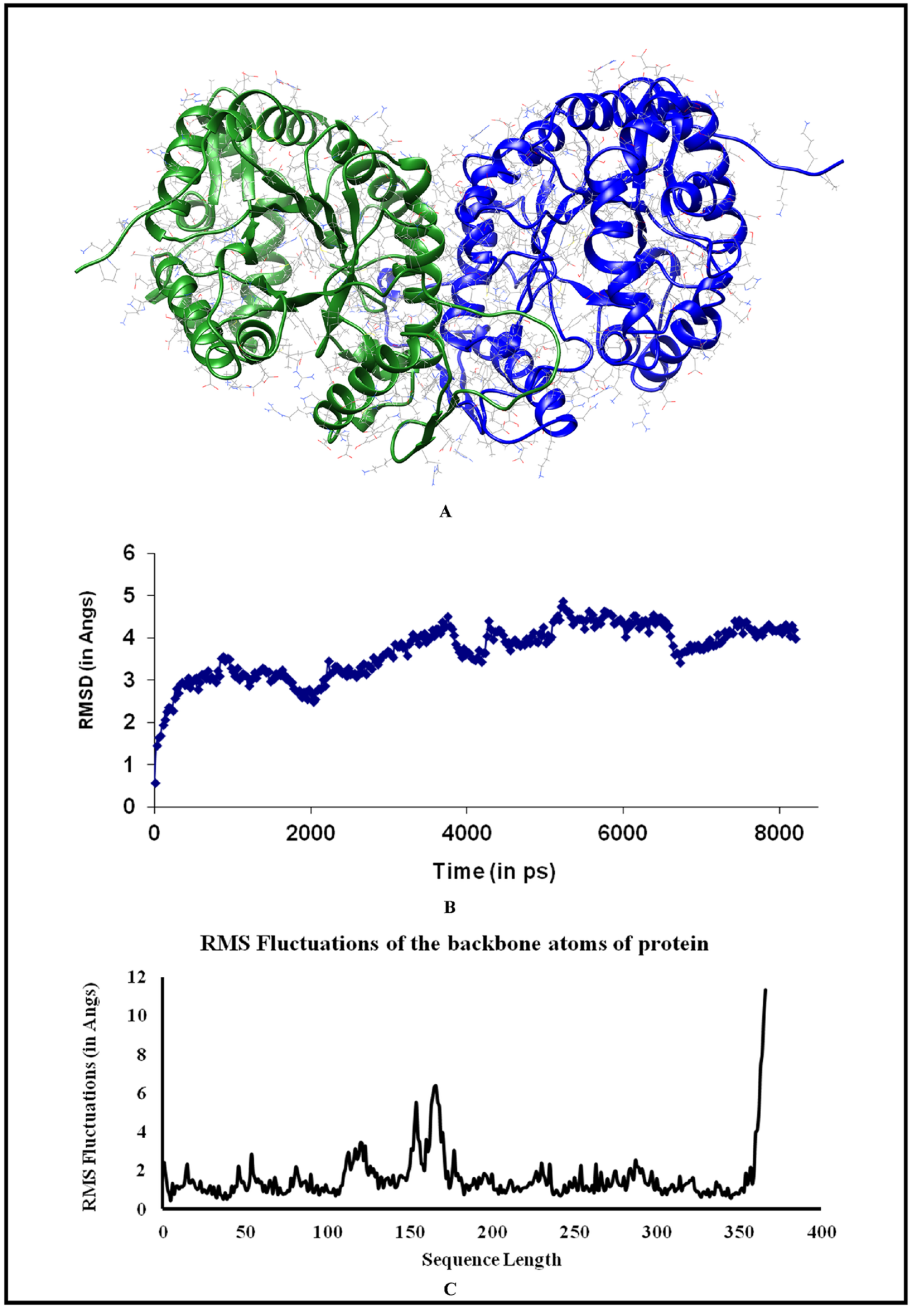
Modeling of MelF structure. (A) Average structure of modeled MelF (in dimeric state). (B) Structural equilibration and atomistic simulation of MelF for 8 ns. (C) RMSF for the residues of MelF protein, which indicates the structural rigidity. Region between 150–175 residues shows considerable flexibility.

Sequence of MelF (Accession Number: gi|54289551.1) was PSI-BLASTed against PDB, and 2WGK with 36% sequence identity and 98% sequence coverage was found [19]. Yet Another Scientific Artificial Reality Application (YASARA) Dynamics and Structure (YASARA Biosciences GmBH, Vienna, Austria) (v 10.12.1), software was used for homology modelling and structural refinement (Fig 1A). The loops were built appropriately using Loop-Builder module of YASARA. The structure was refined using YASARA Dynamics and Structure in a sequential process to remove minor conformational strains by simulating the protein for 500 ps. The refined structure of MelF was validated by WHATIF server (S3 Table). The simulation was further continued using AMBER-03 force field for 8 ns. The average structure was created from snapshots from 5.2 to 8 ns time frame when the modeled structure reached a stable phase. The process of averaging the atomic coordinates also yielded the information about relatively rigid and flexible parts (Fig 1C). The alternative structures were combined into a single representation of the ensemble to account for large scale conformational changes (Fig 1B). The conformational changes were calculated as Root Mean Square Fluctuation (RMSF) as detailed below.

$$RMSF=\sqrt{\frac{1}{T}\overset{T}{\underset{i=1}{\sum }}{\left(V_{i}-\bar{V}\right)}^{2}}$$

Where T = number of frames and V is coordinate set of backbone

The most deviant regions are in between 150–175 residues (Fig 1C). This region (150–175) shows high root mean square fluctuation (rmsf) thus suggesting its less ordered structural configuration. Interestingly, the structural information for residues 175–180 is missing from the crystal structure of pdb entry 5AEC (5AEC replaces PDB entry 2WGK) probably this stretch is highly flexible. None of residues from these flexible regions were part of the docking site. Similar approach was also adopted in our earlier work and by other researchers to process the PDB crystal structure of C2 domain [20,21]. The model was further validated by Whatcheck (S3 Table). The Ramachandran plot for 3D structure of MelF was also drawn (S4 Fig). The validated structure was used to identify a druggable pocket/site using Pocket-finder, Q-site finder [22] and InCaSitefinder [23]. The top-scoring pockets were scrutinized to identify a pocket with better druggability index. One such pocket on MelF was shortlisted as the best druggable pocket.

### Compound filtration and VLS

We used a bimodal approach for screening drug-like compounds, which involved a consensual approach based on DOCK [24] and Vina [25] docking methods. The ChemBridge Corp. screening collection of ~1 million molecules (2013–2014) was selected for *in silico* screening. These were filtered using FAF-Drugs2 [26] with the hard-filters. The remaining ~250,000 compounds that satisfied the ADMETox filters were used for the docking studies. These sdf-formatted files were converted to PDB format using Open Babel 2.2.3 [27]. Following conversion to PDB format, files containing individual compounds were created and processed using MGLTools. DOCK 3.6 and Vina 1.0.2 were used for all protein-ligand docking calculations in this study. The docking parameters for Vina were kept to their default values. The interior of the pocket is an elongated space surrounded by the residues Phe7, Met9, GLu44, His45, Glu51, Met205, Leu207, Tyr268, Val269, Thr272, Phe273, Phe276 Met278, Leu331 and Val240. The dimension of the docking grid was 28 x 21 x 18. The docking site was centered on coordinates (-20.412,-9.0,-61.533). The docked compounds were manually checked to assess their interaction with protein.

We used DOCK 3.6 to screen non-toxic drug-like compounds against the modeled structure of MelF. Each ligand pose was scored as the sum of the receptor-ligand electrostatics (which was corrected for desolvation of ligand). In order to improve charge complementarity between the enzyme and docked molecule, the absolute magnitude of the partial atomic charges of several active site atoms were increased by 0.4 units (the net residue charges were unchanged): Tyr268OH and HOH; Thr272O*γ*1 and HO*γ* and Thr185O*γ*1 and HO*γ*. This method has already been used by Babaoglu *et al* [28]. Conformations were generated using concord. Ligand charges and initial solvation energies were calculated using AMSOL. The top 1000 molecules that were present in ranked lists of both methods were selected as consensual putative inhibitors. Among these, 178 molecules were shortlisted for experimental testing based on their docking score (only top-scoring compounds were tested), scaffold diversity and our assessment of how well they fit in the targeted site.

### Cloning and expression of *M*. *marinum* MelF

*M*. *marinum* strain M genomic DNA sequence (GenBank accession number NC_010612) was used to design primers 5’-ATG GAT CCA TGA TGG AGA TCG GAA TAT TCC TCA TGC-3’ (forward primer) and 5’-CAA GCT TTC ACT TAT CGT CGT CAT CCT TGT AAT CGA CGA TCG CGG CTT CG-3’ (reverse primer) for PCR amplification of *melF*. The pMAL-c5x vector that carries a MBP-tag [29] was used to clone and express *M*. *marinum melF* in *E*. *coli*. The PCR amplified *melF* from *M*. *marinum* genomic DNA and pMAL-c5x were subjected to restriction digestion with BamH1 and HindIII at 37°C and ligated overnight at 16°C with T4 DNA Ligase. The *melF*::pMAL-c5x plasmid DNA was transformed into *E*. *coli* XL1 Blue and selected on LB agar plates with ampicillin at 100 mg/L. The confirmed clone was transformed to *E*. *coli* BL21, inoculated in LB-glucose medium with ampicillin at 100 mg/L and incubated at 37°C for 3–4 h, till the optical density reached ~ 0.6 at 600 nm. The culture was induced with 1mM IPTG at 37°C for 3 h [29]. The over-expression profile of MBP-tagged MelF protein and its solubility was confirmed by SDS-PAGE. The expression was further confirmed by Western blot analysis using rabbit anti-MBP primary (1:10000) and HRP-conjugated anti-rabbit secondary (1:10000) antibodies [29].

### Purification of MBP-tagged MelF protein

The MBP-tagged MelF protein was purified using amylose resin [29]. The IPTG induced *melF*::pMAL-c5x bacteria were harvested, washed and resuspended in 5 times column buffer (CB) (20mM Tris-Cl, pH 7.4, 200mM NaCl, 1mM EDTA). After the addition of lysozyme and phenylmethylsulfonyl fluoride at a final concentration of 1mg/mL and 1mM, respectively, the bacterial cells were kept on ice for 30 min, followed by sonication and centrifugation at 10,000 rpm for 30 minutes at 4°C. The supernatant (WCL) was collected, applied to amylose column for 3–4 h at 4°C and washed with CB. The protein was eluted with an elution buffer (10mM maltose in CB) by collecting 15–20 fractions at 4°C and the presence of purified MelF protein in the collected fractions was checked with 10% SDS-PAGE. The fractions containing MelF were pooled and concentrated to 1mg/mL by Amicon Ultra Centricon with a cutoff value of 30 kDa at 4°C. The purified enzyme was run on HPLC using C-18 column (250 X 4.60 mm) (Shimadzu, Japan) with acetonitrile:water (90:10) as the mobile phase and a flow rate of 0.5 mL/min. The protein was detected at A_280_ using UV-detector. To rule out non-specific mechanism of the compounds as protein aggregators, 100 μg of purified MelF protein was incubated with 30 μM of each inhibitor in 100 μL reaction mixture at 12°C for 15 min and then loaded on native-PAGE, to see, any shift of protein band.

### Flavin oxidoreductase assay

The enzyme activity was assayed by monitoring a decrease in absorption of NADH (nicotinamide adenine dinucleotide) at 340 nm (ε = 6.22 X 10^3^ M^-1^cm^-1^) in 50 mM Tris-Cl, pH 7.4 at 25°C. The steady state kinetic measurements were performed in a 1-cm path length cuvette in a final volume of 1mL according to the method of Kendrew *et al*. [30] with slight modifications. 300μM of NADH and 80μM of FAD (flavin adenine dinucleotide) were added and the assay was initiated with the addition of 100 μg (6.09× 10^−10^ mole) of enzyme present in WCL/purified MelF. The reaction was followed at 25°C for 5 min. The FAD was kept in the dark to prevent photo-reduction. The specific activity of the enzyme was calculated as

$$\begin{array}{cc} \text{Units}/\text{mg } & =\;\frac{\Delta {\text{A}}_{340/\text{min}}}{6.22\times \text{mg enzyme}/\text{ mL reaction mixture }} \end{array}$$

Where ΔA_340_ represents a change in absorbance of NADH at 340 nm.

V_max_ and K_m_ values were determined using different substrate concentrations for 100 μg of purified enzyme by plotting the non-linear regression curve. k_cat_ was calculated from the formula:

$${\text{k}}_{\text{cat}}=\frac{{\text{V}}_{\text{max}}}{{\text{E}}_{\text{t}}}$$

Where k_cat_ = Turnover number (maximum velocity per active site), ${\text{E}}_{\text{t}}\left(\mu \text{mole}\right)=\frac{\text{Wt}\left(\text{g}\right)\text{ X }10^{6}}{\text{Mw}\left(\text{g}/\text{mole}\right)}$

### Screening of inhibitors based on flavin oxidoreductase assay

The inhibitors were dissolved in DMSO to a final concentration of 100 μM in the reaction mixture. The final DMSO concentration in the reaction mixture was ~ 0.3–0.5% and DMSO control was also run (0.5% DMSO, no other additives) to rule out non-specific inhibitory effect. A preliminary screening of 178 inhibitors was carried with the IPTG induced WCL and 20 shortlisted compounds were further tested with the purified MelF. The percent enzyme activity in presence of inhibitors was calculated as

$$\text{\% enzyme activity }=\frac{\Delta {\text{A}}_{\text{compound}}}{\Delta {\text{A}}_{\text{control}}}\times 100$$

ΔA_compound =_ change in absorbance in presence of inhibitor and ΔA_control_ = change in absorbance of control, the enzyme activity of WCL/purified protein in absence of inhibitor. The enzyme activity of uninduced WCL was subtracted from the induced WCL to get the actual enzyme activity. To prevent auto-oxidation of NADH, all the tests were carried out in cuvettes covered with parafilm. A control was also run in which no enzyme was added to ensure that the decrease in absorbance was not due to self-oxidation of NADH.

### K_i_ (inhibitory constant) determination

K_i_ values were calculated using Dixon plots, where inverse of V_max_ values were plotted against the inhibitor concentration using different substrate concentrations.

### Circular dichroism (CD) spectroscopy

For CD spectral analysis (JASCO Corp, Japan), 0.5 mg/mL of MelF protein was used. A change in the secondary structure of protein was monitored with and without inhibitors in far-UV region between 190–260 nm [31]. The percentage of α-helices and β-sheets were determined using K2D3 software (http://cbdm-01.zdv.uni-mainz.de/~andrade/k2d3/).

### MIC and MBC determination

MIC of 20 inhibitors was determined for *M*. *marinum* and *M*. *tuberculosis* by a high throughput resazurin microtiter plate assay (REMA). Briefly, 100 μL of M-ADC-TW broth was dispensed/well of sterile 96-well microtiter plate (Nunc) and serial two-fold dilutions of each inhibitor were prepared directly in the plate from 1 mg/mL stock solution. To it, 100 μL of bacteria (~ 5 X 10^6^ CFU/well) and 10 μL of resazurin solution (5 mg/mL) were added. The inhibitor control (no inhibitor) and sterile medium control were also included. The plates were incubated at 33°C for 48 h, and 37°C for 96 h, for *M*. *marinum* and *M*. *tuberculosis*, respectively. The concentration of inhibitors that prevented a change in color from purple to pink was taken as an indicator to determine the MIC value [32].

The *in vitro* growth interaction of bacteria with the inhibitors and the first line drugs such as rifampicin (RIF)/INH was determined by resazurin drugs combination microtiter assay [33]. Two-fold serial dilutions ranging from 12.5 mg/L to 0.04 mg/L for RIF/INH were combined with the same concentration of inhibitor (ranging from 12.5 mg/L to 0.04 mg/L) in wells of microtiter plates followed by the addition of bacteria and resazurin solution as described above. The fractional inhibitory concentration index (FICI) was used to evaluate the synergistic effects. The FIC_index_ (FICI) was calculated as follows:

$$\text{FICI }={\text{ FIC}}_{\text{A}}+{\text{FIC}}_{\text{B}}$$

Where

$${\text{FIC}}_{\text{A}}=\frac{\text{MIC of drug A in combination}}{\text{MIC of drug A alone}}$$

$${\text{FIC}}_{\text{B}}=\frac{\text{MIC of drug B in combination}}{\text{MIC of drug B alone}}$$

The effects of the drug combinations were classified as synergistic (FICI ≤ 0.5), additive or no interaction (FICI > 0.5–4), and antagonistic (FICI > 4) as described by Pagliotto et al [33].

The MBC microtiter plates were prepared similar to REMA plates, but no resazurin was added [32]. For MBC determination, the plates were incubated at 33°C for 48 h, and 37°C for 96 h, for *M*. *marinum* and *M*. *tuberculosis*, respectively. Then, 100 μL from each well of plates was transferred to a plate containing 100 μL/well of inhibitor-free M-ADC-TW broth. Ten-fold serial dilutions from each well of MBC plate were plated on 7H10 (M-ADC) agar for CFU enumeration. The MBC was defined as the lowest concentration that resulted in killing of ≥ 99% of the viable bacteria in comparison with the starting inoculums. The inhibitor was considered bactericidal when the MBC/MIC ratio was ≤ 4 [32]. To determine the enhanced bactericidal effect, combinations of drugs (RIF/INH) and inhibitors were used as stated above. Briefly, two-fold serial dilutions ranging from 12.5 mg/L to 0.04 mg/L for RIF/INH were combined with the same concentration of inhibitor in wells of microtiter plates followed by the addition of bacteria as described above. The enhanced bactericidal effect was considered when the MBC/MIC ratio of combination was lesser as compared to RIF/INH alone. The MIC/MBC experiments were run in triplicate to ensure the reproducibility.

### Cytotoxicity assay

The MTT (3-(4, 5-dimethyl thiazol-2yl)-2, 5-diphenyl tetrazolium bromide) assay was employed to assess the cytotoxicity of the inhibitors [34,35]. HeLa cells (Human cervical epithelial cells) were plated at a concentration of 1×10^5^/well in 100 μL of minimal essential medium (MEM) supplemented with 10% heat-inactivated fetal bovine serum (FBS) in 96-well plates. After overnight incubation at 37°C in 5% CO_2_ incubator, media was removed and the inhibitors were added in 100 μL of MEM + 10% heat-inactivated FBS at 1X and 5 X MIC concentrations. After incubation for 20 h, the cells were washed thrice with warm MEM (without FBS) and 200 μL of MTT (5mg/mL) was added. The plates were further incubated for 4 h, followed by the addition of 50 μL of DMSO (solubilizing reagent) per well, mixed thoroughly by a micropipette and left for 45 s. Presence of viable cells was visualized by the development of purple color due to formation of formazan crystals. The OD values were read at 595 nm using DMSO as a blank. A standard graph was plotted between the concentration of inhibitors and the relative cell viability.

$$\text{Cell viability }\left(\%\right)\;=\;\frac{\text{Test OD }}{\text{Control OD}}\text{X 100}$$

### Statistical analysis

The student’s t-test was applied to find the difference in the mean enzyme activity ± SD of control and those treated with inhibitors (done in duplicate). V_max_ and K_m_ values were calculated along with 95% confidence interval (CI) by plotting a non-linear regression curve and the analysis was done using Stata 14.2. The P < 0.05 was considered statistically significant.

## Results

### Homology modeling of *M*. *marinum* MelF structure

**Fig 2:**
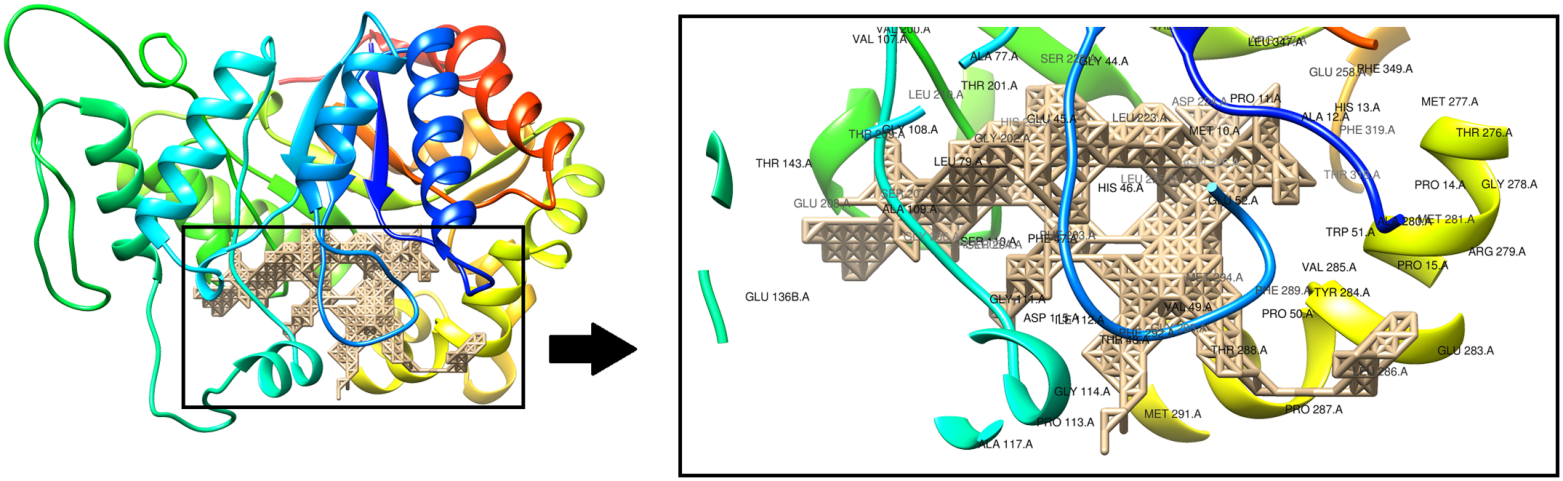
Enlarged view of targeted site for inhibiting MelF oxidoreductase activity. The site shown in grey wire mesh was selected as consenual site; as predicted by InCaSiteFinder, Q-SiteFinder and PocketFinder.

2WGK is a Type II Baeyer-Villiger monooxygenase from *Pseudomonas putida* [19], which has sequence coverage of ~ 98% and sequence identity of ~ 36%, with *M*. *marinum* MelF protein. Since there was a high sequence similarity between the MelF protein and 2WGK, we reasoned that a reliable homology model could be constructed. 2WGK is an FMN-dependent dimeric enzyme; therefore, *M marinum* MelF was modeled as a dimeric structure. Following an equilibration, the three-dimensional (3D) modeled structure of MelF protein was refined by all-atom molecular dynamics simulation by optimizing its energy parameters and removing the structural strains (Fig 1B and 1C). The average MelF structure (Fig 1A) thus derived was checked by Whatif server for its quality. The average structure was found to be in good harmony with the set parameters for a good structure with RMSD in bond angles 1.509 (S3 Table). A consensus approach was later used to find the best druggable pocket in this structure. The largest pocket providing the most number of contact atoms (630 atoms) and a site volume of 2093 Å^3^ were selected for VLS study (Fig 2, S2 Fig). In addition, the sequence of *M*. *marinum* MelF was compared with *M*. *tuberculosis* H_37_Rv MelF, which showed ~ 98% sequence identity (S3 Fig). Therefore, the proteins encoded by both the sequences are likely to be similar in structure and function independent of their origin.

### Virtual screening

The ChemBridge Core library and Express-Pick Library are two mutually exclusive libraries of commercially available small drug-like compounds at the Hit2Lead.com online store of ChemBridge site. We selected these libraries as they provide a large set of diverse groups of compounds, which is prerequisite to carry out a large scale VLS. 1000 top ranking compounds obtained from Vina (semi-flexible VLS) and DOCK were pooled together. The docked compounds were later manually evaluated to choose molecules and shortlisted for experimental testing based on their docking score (only top-scoring compounds were tested), our assessment of how well they fit the site and their chemical diversity. Diversified set of interacting molecules were clustered and ranked based on structural, energetic and interaction mode parameters. There was a massive overall reduction in the complexity of the chemical space.

**Fig 3:**
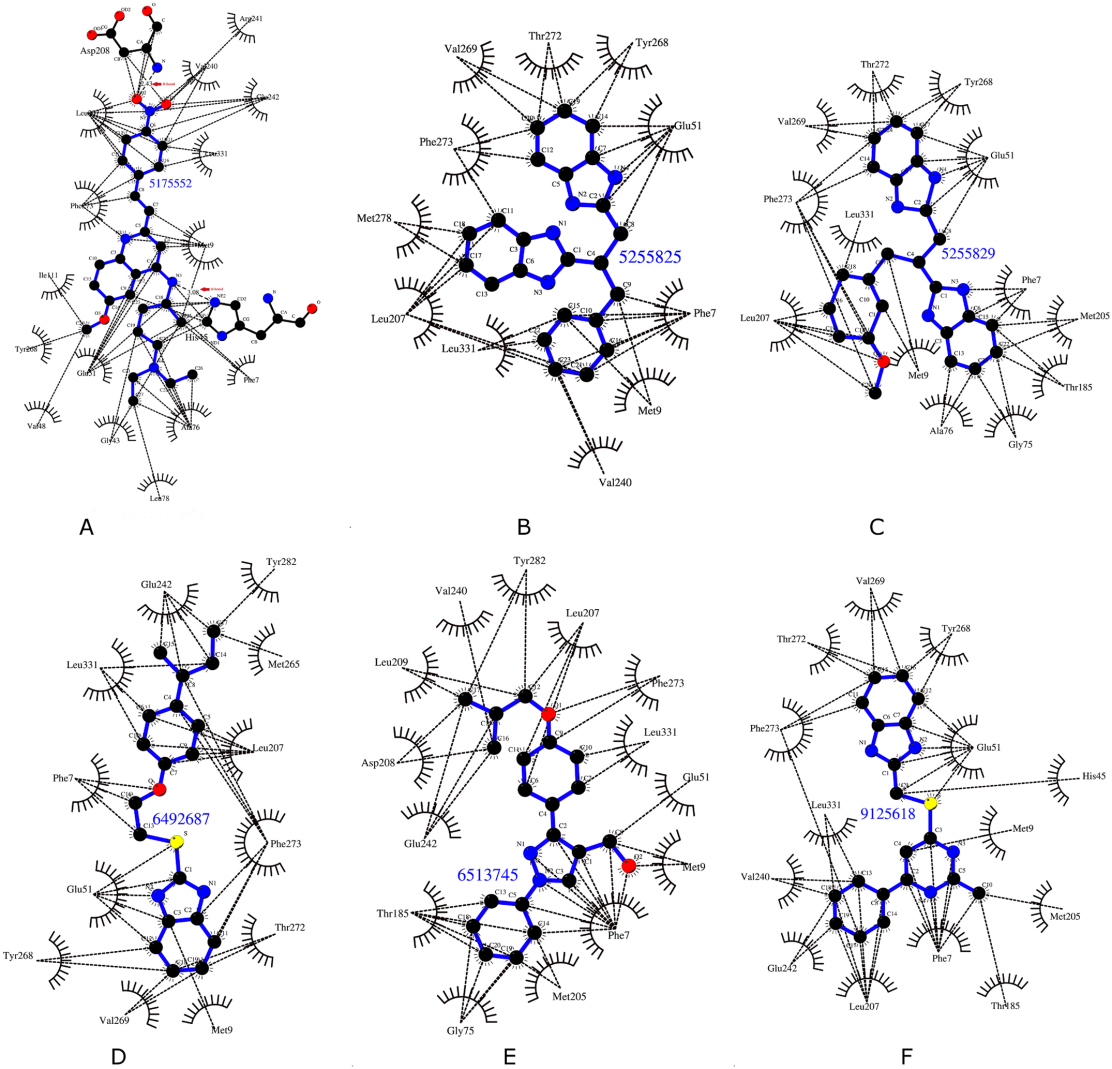
Schematic types of interactions of six compounds with MelF enzyme generated using ligplot. Interaction of MelF with (A) # 5175552 (B) # 5255825 (C) # 5255829 (D) # 6492687 (E) # 6513745 (F) # 9125618. The compounds showed a predominance of van der Waals interactions. Hydrogen bonds are labeled (shown with red arrows), whereas dashed lines (------) display van der Waals interaction.

**Fig 4:**
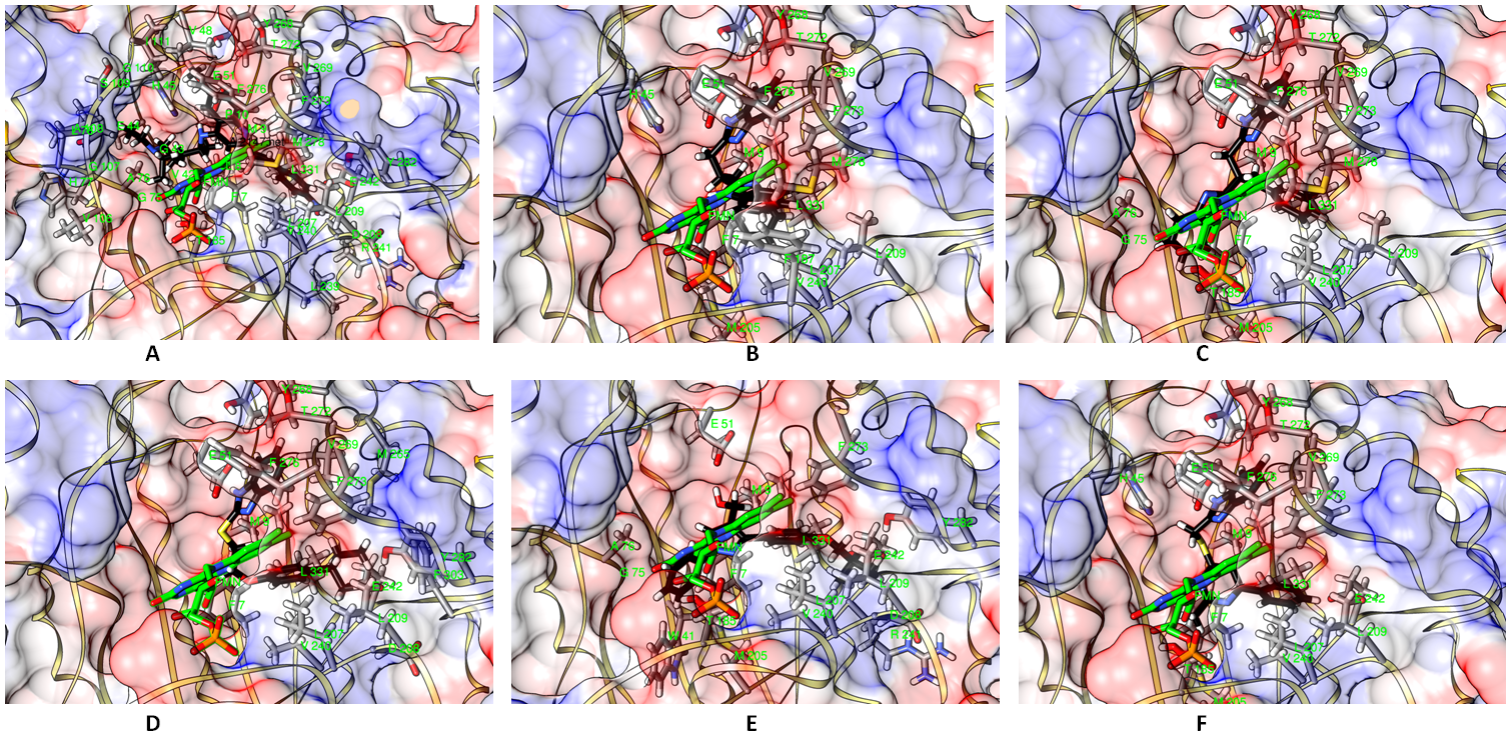
Post-docking interactions between targeted site residues of MelF protein with compounds. The protein is depicted in transparent surface view, whereas FMN (green sticks for carbon atoms) and compounds [black sticks for carbon atoms; (A) # 5175552 (B) # 5255825 (C) # 5255829 (D) # 6492687 (E) # 6513745 (F) # 9125618] within the binding pocket. The interacting amino acids are also shown as sticks (grey color for carbon atoms). Rests of the atoms are colored as per convention.

Among the top six top compounds, compound # 5175552 made multiple interactions with MelF. N atom of Asp208 formed a hydrogen bond with the O2 of the compound; NE2 atom of His45 formed a hydrogen bond with N3 of the compound and the multiple hydrophobic interactions occurred between Phe7, Met9, Ala76, Ile111, Leu207 and Phe273 with the aromatic rings of compound # 5175552. Similarly, compound # 6492687 exhibited numerous identical contacts vis-à-vis compound # 5175552. Of the 14 amino acids that interacted with compound # 9125618, 10 amino acids (Phe7, Met9, His45, Glu51, Leu207, Val240, Glu242, Phe273, Ley331 and Tyr268) were common with the interacting residues of compound # 5175552. (Fig 3A, 3D and 3F). The compound # 5255825 and # 5255829 were structurally similar and this was also reflected somewhat in the binding modes (Fig 3B and 3C). The docked poses of compounds displayed partial occupancy at the same site as that of FAD/FMN (Fig 4A–4F). As per the docking poses, compound # 5255825 interacted with Phe7, Met9, Glu51, Val240, Leu207, Met278, Phe273, Val269, Tyr268, Thr272 and Leu331. About 80% contacts between compound # 5255829 and MelF residues were similar to those observed with compound # 5255825. Interactions made by compound # 9125618 and # 651374 were primarily hydrophobic in nature (Fig 3E and 3F). MelF residues interacting with compound # 6492687 and # 6513745 considerably overlapped amongst themselves as 8 out of 13 amino acids that interacted with compound # 6513745 also interacted with the compound # 6482687. Interestingly, *in silico* poses of the compounds exhibited a predominance of van der Waals interaction with MelF (Fig 3). Moreover, as per the docking results, the compound # 6492687 and # 6513745 overlapped considerably with the FAD/FMN interacting site as compared to compound # 5255825 and # 5255829 (Fig 4B–4E).

### Cloning, expression and purification of MelF protein

**Fig 5:**
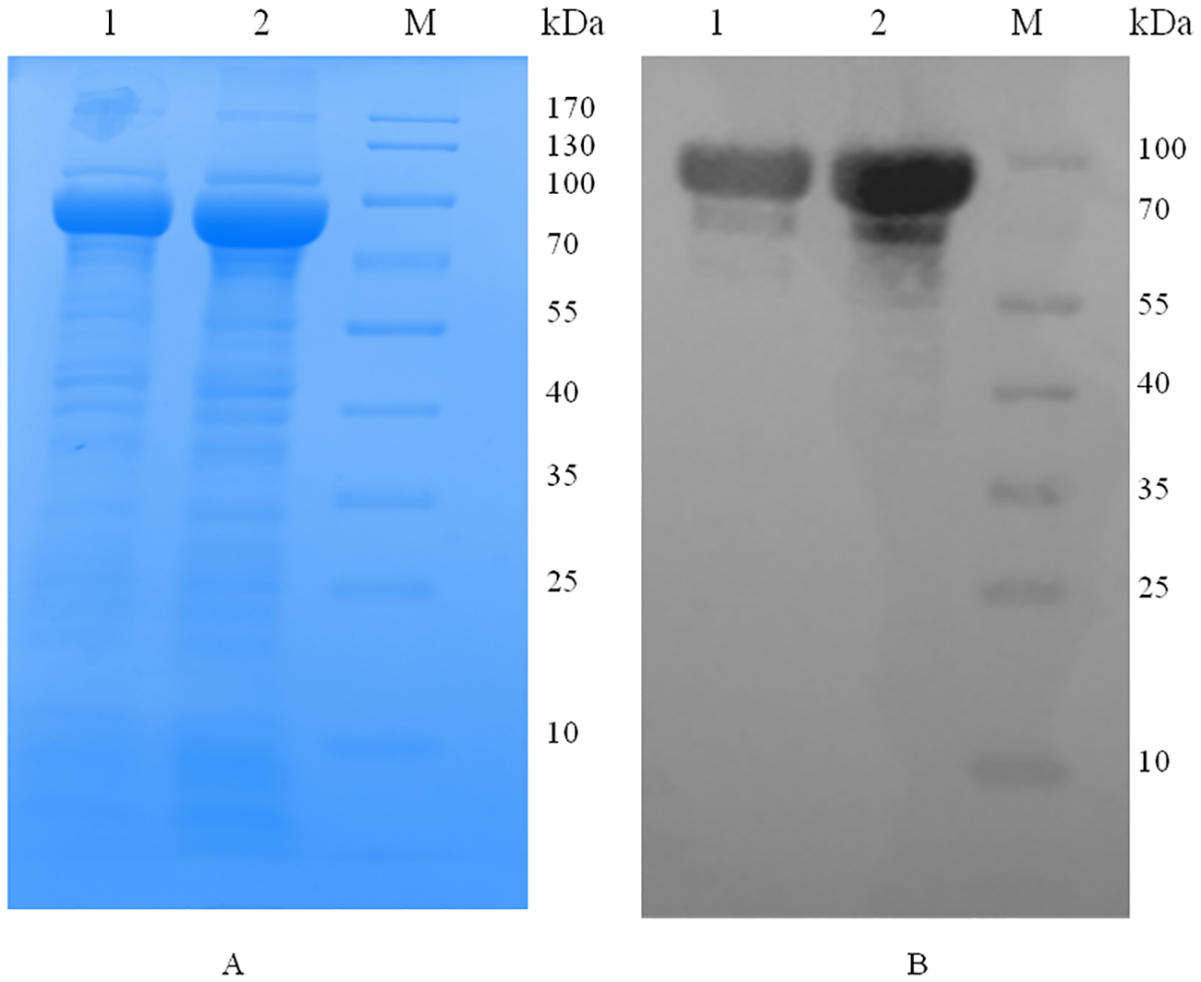
Expression and purification of MelF protein using pMAL-c5x vector. (A) SDS-PAGE showing over-expressed MBP-tagged MelF protein for WCL (lane 1) and whole bacterial suspension (lane 2). (B) Western blot analysis for WCL (lane 1) and whole bacterial suspension (lane 2) for over-expressed MBP-tagged MelF Protein; M represents protein marker.

**Fig 6:**
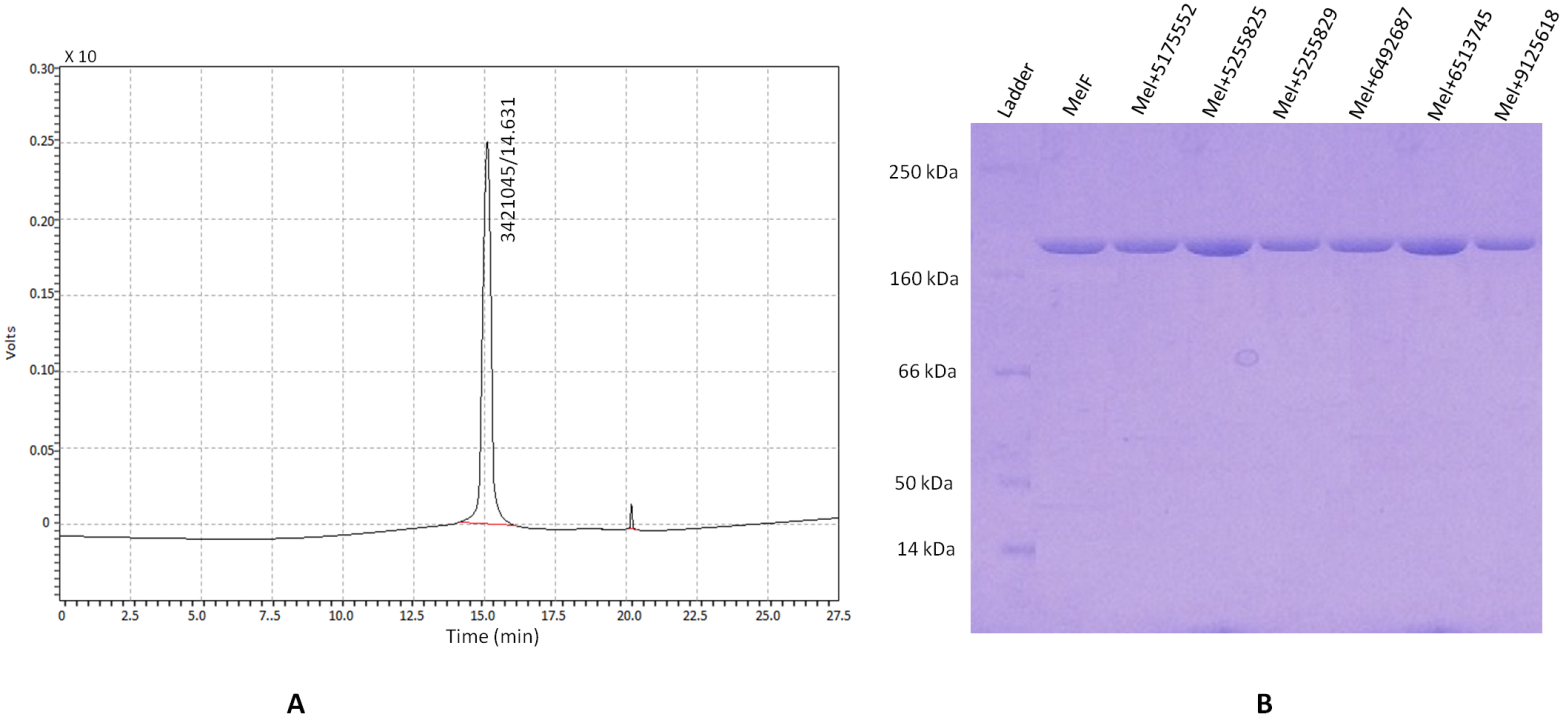
(A) Analysis of purified MelF by HPLC shows a major peak of MelF; 3421045 represents peak area, while 14.631 represents retention time. (B) Native-PAGE of purified MelF alone and in combination with inhibitors; Ladder represents protein markers.

The *melF* clones were tested via fragment release by BamH1 and HindIII (S1 Fig). The *melF* was inserted downstream from the *malE* of *E*. *coli*, which encodes for MBP thus resulting in the expression of MBP-MelF fusion dimeric protein. The over-expression of MelF protein was visualized on a 10% SDS-PAGE gel with a distinct band of ~ 82 kDa of MBP-MelF fusion protein on SDS-PAGE (~ 42kDa of MelF plus ~ 40kDa of MBP) and ~164 kDa (~ 84 kDa MelF + ~ 80 kDa MBP) on native-PAGE in the induced WCL (Figs 5A and 6B). The soluble nature of the protein was established with SDS-PAGE (Fig 5A) as most of the expressed protein was present in the WCL (soluble fraction) and ~ 40% of the total bacterial protein was MelF. Western blot analysis revealed a clear visible band with WCL and the whole bacterial suspension (Fig 5B) in a chemiluminescent assay thus confirming that the over-expressed protein was MBP-MelF fusion protein. Since MBP has a strong affinity for tighter binding to amylose, MelF protein was purified using MBP’s affinity for maltose in a one-step purification process thus indicating that MelF was properly translated and folded in *E*. *coli*. The purified MelF (Fig 6A) could display a sharp peak at 280 nm by HPLC. To rule out non-specific mechanism of the compounds as protein aggregators, we performed native-PAGE analysis in the presence and absence of inhibitory compounds (Fig 6B). Notably, the inhibitors did not promote protein aggregation as there was no shift in the bands of MelF in their presence.

### Enzyme kinetics and screening of inhibitors

**Fig 7:**
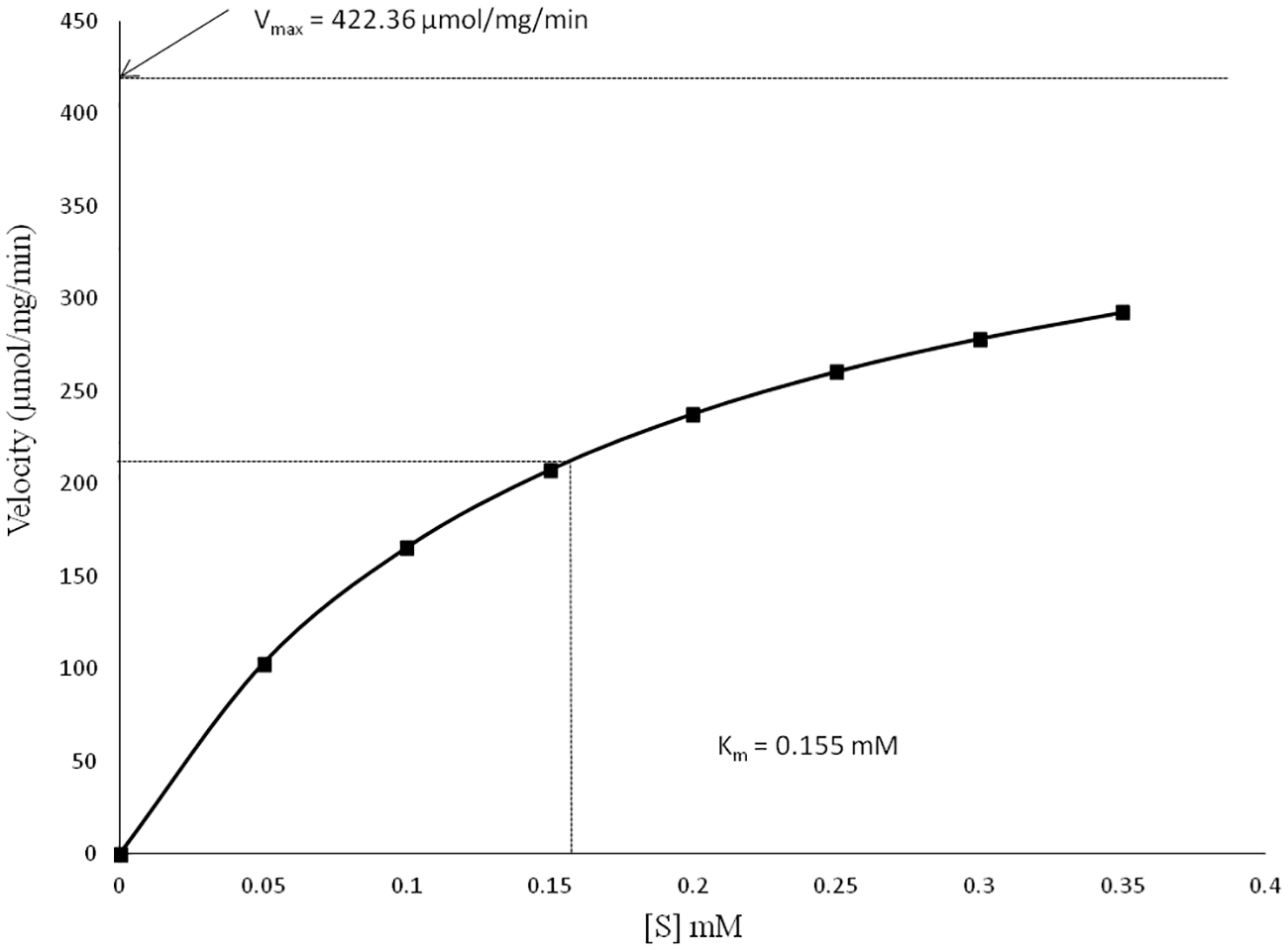
Determination of V_max_ and K_m_ for MelF flavin oxidoreductase by non-linear regression curve. The coefficient of variation (R^2^) was 0.986 and the corresponding equation was y = -2872.2x^2^ + 1808x. The data represent the mean of three independent observations.

The specific activity of flavin oxidoreductase of purified MelF was somewhat higher in comparison to WCL (Table 1). The low difference between the specific activities of enzyme from WCL and the purified form could be due to the presence of other oxidoreductases present in WCL with a similar reaction. V_max_ and K_m_ of the purified MelF were determined to be 422.36 μmole/min/mg (95% CI, 301.7–543 μmole/min/mg) and 0.155 mM (95% CI, 0.05–0.26 mM), respectively, whereas k_cat_ value of the purified MelF was determined to be 6.94 X 10^5^ min^-1^ (Fig 7).

**Table 1:** Flavin dependent oxidoreductase activity with WCL and purified MelF.

Sample	Activity* (μmole/min)	Specific Activity* (μmole/min/mg)
Induced WCL	17.6	176
Purified MelF	22.18	221.8

*The substrate concentration was 300 μM and enzyme concentration was 100 μg (6.09 × 10^−10^ mole).

**Fig 8:**
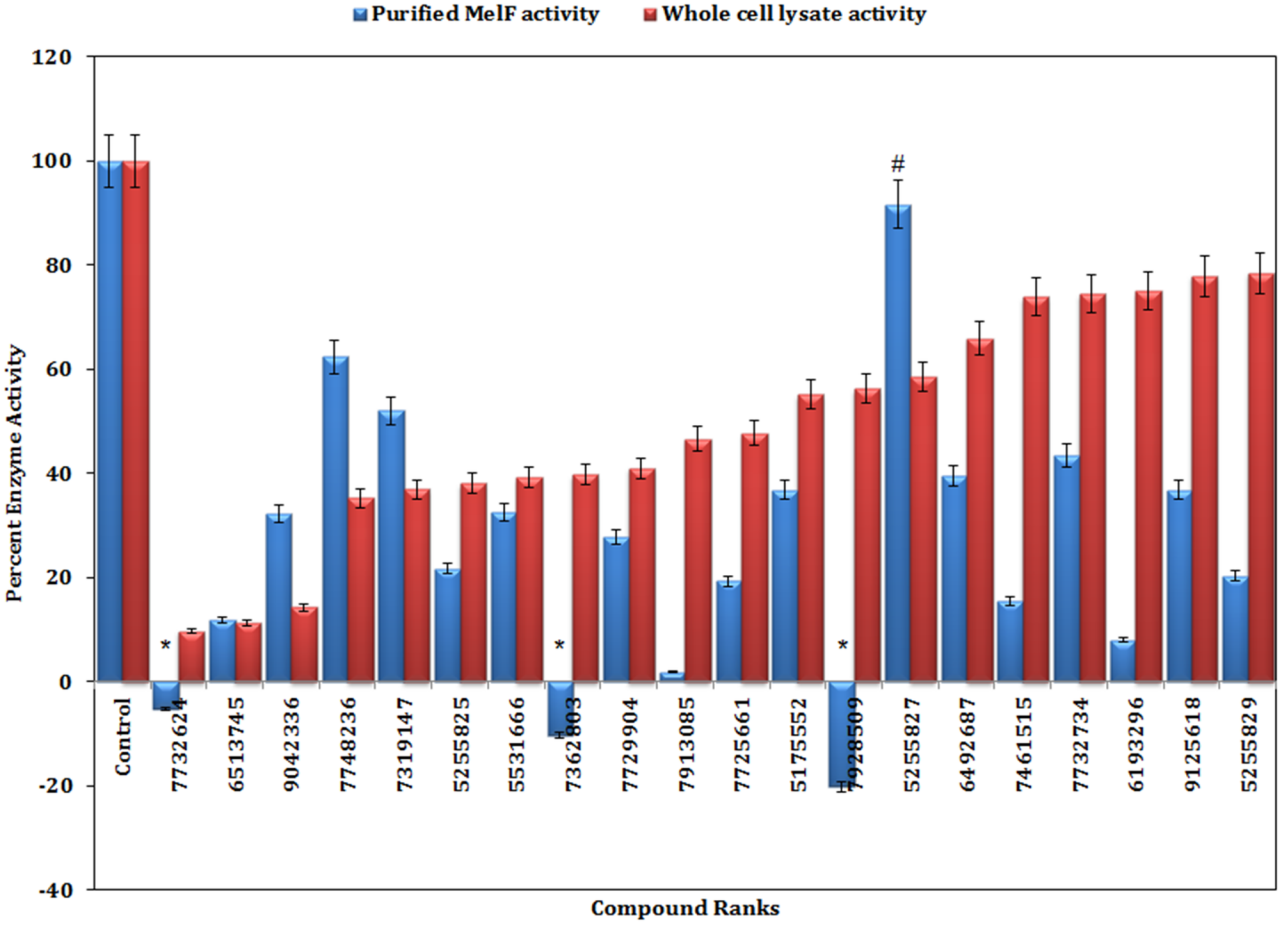
Change in flavin oxidoreductase activity of the purified MelF (blue bars) and WCL (red bars) in presence of inhibitors as compared to control (enzyme activity in the absence of inhibitors). Values were mean ± SD of two replicates. Among 20 inhibitors tested, 16 showed significant inhibition (P < 0.05) in enzyme activity in comparison to control; while inhibitors ^#,*^ revealed no inhibitory effect (P > 0.05).

While screening a total of 178 compounds with WCL, 20 were shortlisted, which displayed significant inhibition (P < 0.05) in the enzyme activity ranging from 22% to 90% (Fig 8). Such compounds were further tested to inhibit enzyme activity of the purified MelF and that was found to be greater than WCL. Out of 20 compounds, 16 were found to significantly inhibit (P < 0.05, Fig 8) the enzyme activity of purified MelF (38–98% inhibition). On the other hand, the compound # 5255827 did not inhibit (P > 0.05) the enzyme activity of purified MelF (with residual enzyme activity of ~ 100%), though its inhibitory effect was observed in WCL. As the total protein concentrations as well as the compound concentrations in WCL and the purified MelF were equal, therefore, the effective concentration of MelF in WCL was relatively lower than the purified MelF. The compound concentration in WCL was probably adequate to inhibit the MelF activity (owing to less effective concentration of enzyme); however, this effect of compound was abolished when the enzyme concentration was increased (as expected in the purified MelF). Another possibility could also be that the compound # 5255827 might be inhibiting oxidoreductases other than MelF in WCL. Moreover, the virtual screening is not 100% accurate implying that some of the inhibitors designed might be interacting with the target(s) other than MelF.

In addition, three compounds (# 7732624, # 7362803, # 7928509) that earlier showed an enzyme inhibition with WCL could not display such inhibitory effect with the purified MelF. In fact, these compounds displayed an enhanced absorbance values at 340 nm in comparison to control. Perhaps, the increase in absorbance does not necessarily mean stimulation of the enzyme activity as these compounds might be getting altered during oxidation of NADH to NAD^+^ thus leading to enhanced absorbance. However, in-depth investigation is required to rule out this possibility.

**Fig 9:**
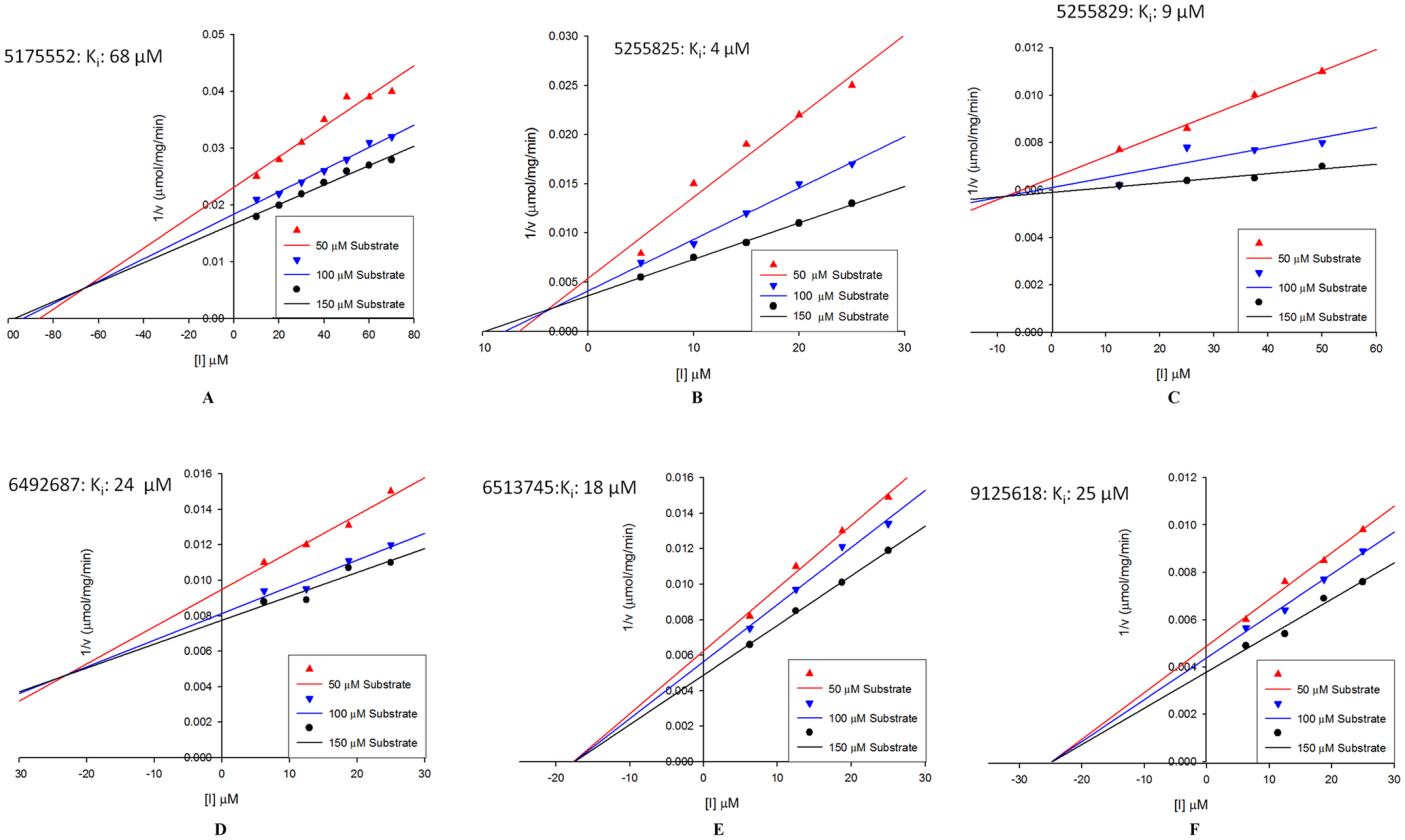
Dixon’s plots for K_i_ calculation. Velocity was calculated at three different substrate concentrations (50, 100 and 150 μM) using different inhibitor concentrations.

The intersection of Dixon’s plot at all values of [S] were not on [I] axis for inhibitor # 5175552, # 5255825, # 5255829 and # 6492687 (Fig 9), which suggests competitive inhibition. On the contrary, intersection of these plots for inhibitor # 6513745 and # 9125618 was on [I] axis at [I] = -K_i_ thus suggesting noncompetitive inhibition. Furthermore, inhibitor # 5255829 and # 5255825 displayed K_i_ value of 9 μM and 4 μM respectively. These were followed by inhibitors # 6513745, # 9125618 and # 6492687 with K_i_ values of 18, 24 and 25 μM respectively, whereas inhibitor # 5175552 was the least effective with K_i_ value of 68 μM (Fig 9, S5 Fig).

**Fig 10:**
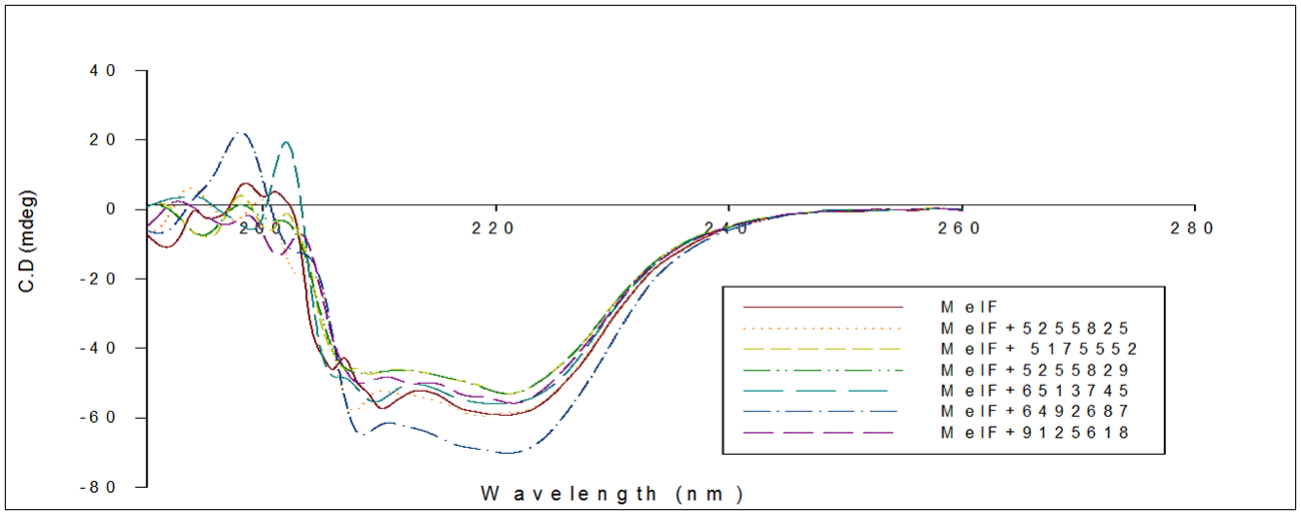
Secondary structural analysis of purified native MelF protein in presence and absence of inhibitors by CD spectra.

The secondary structure of protein was determined using its respective CD spectra. The α-helices and β-sheets were almost constant with and without inhibitors (Fig 10, S2 Table) thus suggesting that these compounds are not denaturants.

### MIC and MBC determination

20 compounds shortlisted on the basis of enzyme inhibition were subjected to growth inhibition assay and MIC was determined by REMA against *M*. *marinum* and *M*. *tuberculosis*. Notably, six compounds (Table 2) showed an MIC value ranging from 3.38–19.39 μM, and 13.51–38.77 μM, for *M*. *marinum* (at 48 h) and *M*. *tuberculosis* (at 96 h), respectively. Of these, the compound # 5175552 showed an MIC of 3.38 μM and 13.51 μM for *M*. *marinum* and *M*. *tuberculosis*, respectively. However, five compounds (# 6492687 # 6513745, # 5255825, # 9125618, # 5255829) displayed an MIC of 9.57–19.39 μM, and 32.68–38.77 μM, for *M*. *marinum* and *M*. *tuberculosis*, respectively.

**Table 2:** MIC and MBC determination of top six compounds against *M*. *marinum* and *M*. *tuberculosis* (~ 5 X 10^6^/well) by REMA and CFU assay, respectively.

Drug	*M*. *marinum*			*M*. *tuberculosis*		
	MIC (μM)*	MBC (μM)*	MBC/MIC	MIC (μM)*	MBC (μM)*	MBC/MIC
5175552	3.38 (3.38)	6.76 (6.76)	2	13.51 (13.51–27.02)	27.02 (13.51–27.02)	2
5255825	17.74 (8.87–17.74)	35.47 (17.74–35.47)	2	35.47 (17.74–35.47)	35.47 (35.47)	1
5255829	16.30 (8.15–16.30)	32.68 (32.68)	2	32.68 (32.68)	32.68 (32.68)	1
6492687	9.57 (9.57)	38.28 (38.28)	4	38.28 (19.14–38.28)	38.28 (38.28)	1
6513745	19.39 (9.69–19.39)	38.77 (19.39–38.77)	2	38.77 (19.39–38.77)	38.77 (19.39–38.77)	1
9125618	18.80 (9.40–18.80)	37.61 (37.61)	2	37.61 (18.80–37.61)	37.61 (37.61)	1
RIF	0.475 (0.475)	0.95 (0.47–0.95)	2	0.048 (0.048–0.095)	0.095 (0.048–0.095)	2
INH	4.53 (2.27–4.53)	4.53 (4.53)	1	2.27 (2.27)	2.27 (2.27)	1

RIF, Rifampicin; INH, Isoniazid;

*Results are expressed as the median and range (minimum-maximum) of three independent experiments (Andreu et al., 2012).

The six best compounds were further tested for MBC determination by the conventional CFU assay (Table 2). The compound # 5175552 showed an MBC of 6.76 μM and 27.02 μM, with an MBC/MIC ratio of 2.0, for *M*. *marinum* and *M*. *tuberculosis*, respectively. On the other hand, the other five compounds revealed an MBC of 32.68–38.77 μM for both *M*. *marinum* and *M*. *tuberculosis*. These five compounds exhibited an MBC/MIC ratio of 2.0–4.0 and 1.0–2.0 for *M*. *marinum* and *M*. *tuberculosis*, respectively. Notably, all the six compounds showed bactericidal activity against *M*. *marinum* and *M*. *tuberculosis* as MBC/MIC ratio was ≤ 4. The combined inhibitory effect of these compounds in combination with RIF/INH was also examined (Table 3). It was found that three compounds (# 5175552, # 5255825 and # 6492687) showed synergistic inhibitory effect in combination with INH against *M*. *tuberculosis* with FICI of 0.257–0.263, whereas two compounds (# 5255825 and # 6513745) showed such synergistic effect against *M*. *marinum* with FICI of 0.276. Furthermore, two compounds (#5175552 and # 9125618) also displayed an enhanced bactericidal effect for *M*. *tuberculosis* in combination with RIF (Tables 2 and 3) as two-fold lesser MBC/MIC ratio was observed in comparison to RIF alone.

**Table 3:** Combined effect of drugs with inhibitors against *M*. *marinum* and *M*. *tuberculosis*.

Drug + Inhibitor	*M*. *marinum*				*M*. *tuberculosis*			
	MIC (μM)*	MBC (μM)*	MBC/MIC	FICI	MIC (μM)*	MBC (μM)*	MBC/ MIC	FICI
RIF+ 5175552	0.66 (0.66–1.32)	1.32 (1.32–2.64)	2	0.629	0.066 (0.066–0.132)	0.066 (0.066–0.132)	1	0.503
RIF+ 5255825	0.791 (0.791)	1.582 (1.582)	2	0.536	0.079 (0.079–0.158)	0.158 (0.158)	2	0.502
RIF+ 5255829	0.746 (0.746–1.492)	1.492 (1.492–2.984)	2	0.530	0.094 (0.094–0.188)	0.188 (0.188)	2	0.627
RIF+ 6492687	0.835 (0.835)	1.67 (1.67)	2	0.561	0.105 (0.105)	0.21 (0.21)	2	0.627
RIF+ 6513745	0.843 (0.843–1.686)	1.686 (1.686–3.372)	2	0.530	0.106 (0.106)	0.21 (0.21)	2	0.627
RIF+ 9125618	0.825 (0.825)	1.65 (1.65)	2	0.530	0.103 (0.103)	0.103 (0.103–2.06)	1	0.627
INH+ 5175552	2.95 (1.47–2.95)	2.95 (2.95)	2	0.701	0.737 (0.737–1.474)	1.474 (0.737–1.474)	2	0.263
INH+ 5255825	1.582 (1.582–3.164)	3.164 (1.582–3.164)	2	0.276	0.791 (0.791)	0.791 (0.791–1.582)	1	0.257
INH+ 5255829	3.094 (3.094)	3.094 (1.547–3.094)	1	0.552	1.547 (1.547)	1.547 (1.547)	1	0.514
INH+ 6492687	3.212 (3.212)	3.212 (3.212)	1	0.602	0.808 (0.808–1.616)	0.808 (0.808–1.616)	1	0.257
INH+ 6513745	1.624 (1.624)	1.624 (1.624)	1	0.276	1.624 (1.624)	1.624 (1.624)	1	0.514
INH+ 9125618	3.22 (1.61–3.22)	3.22 (1.61–3.22)	1	0.552	1.61 (1.61)	1.61 (1.61)	1	0.514

RIF, Rifampicin; INH, Isoniazid;

*Results are expressed as the median and range (minimum-maximum) of three independent experiments (Andreu et al., 2012)

**Fig 11:**
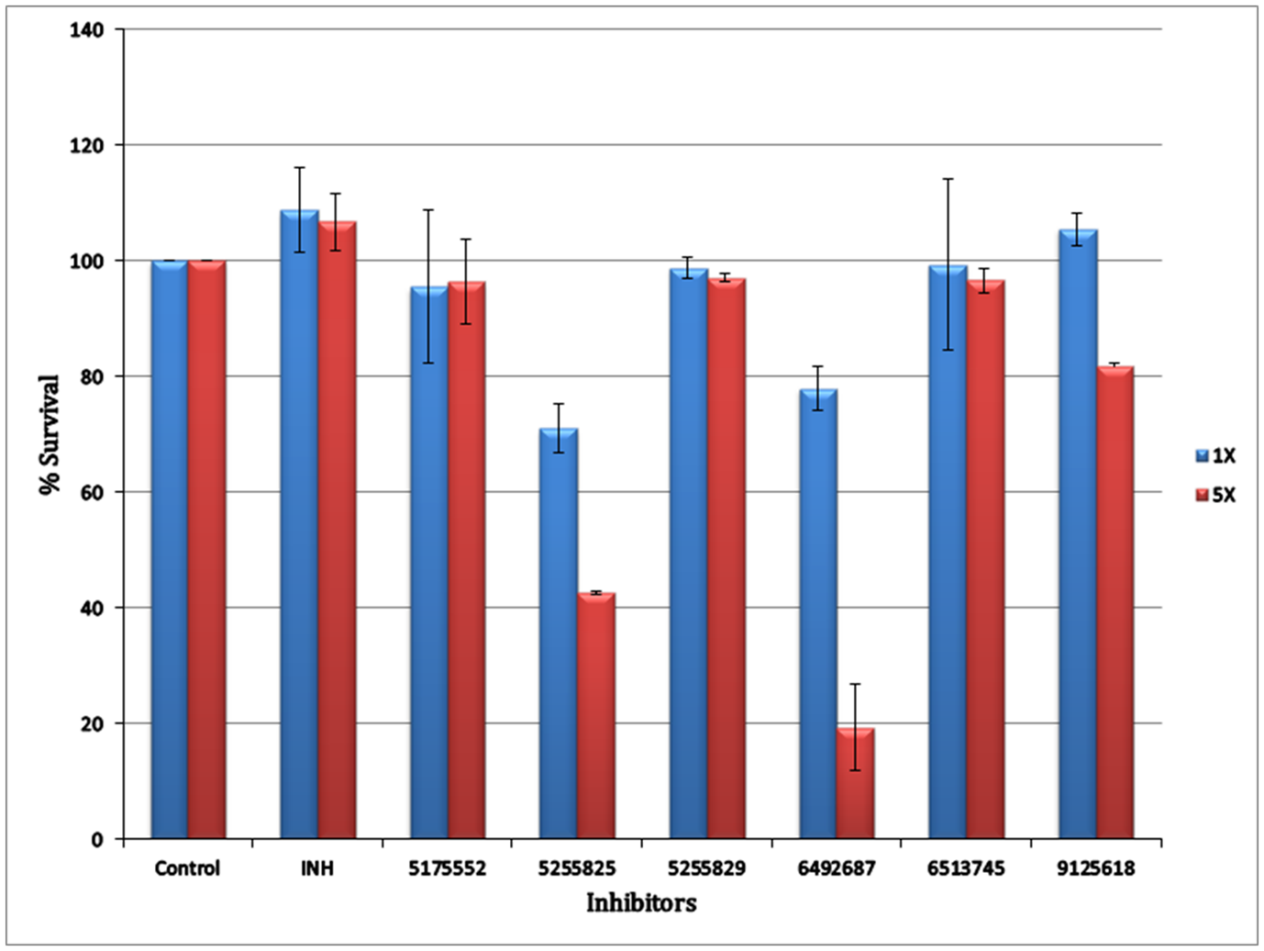
Determination of cytotoxicity of the inhibitors by MTT cytotoxicity assay. Survival of HeLa cells in the presence of 1X and 5X MIC concentrations of inhibitors. INH was taken as a positive control. The experiments were done in duplicate and the data was represented as mean ± SD.

**Fig 12:**
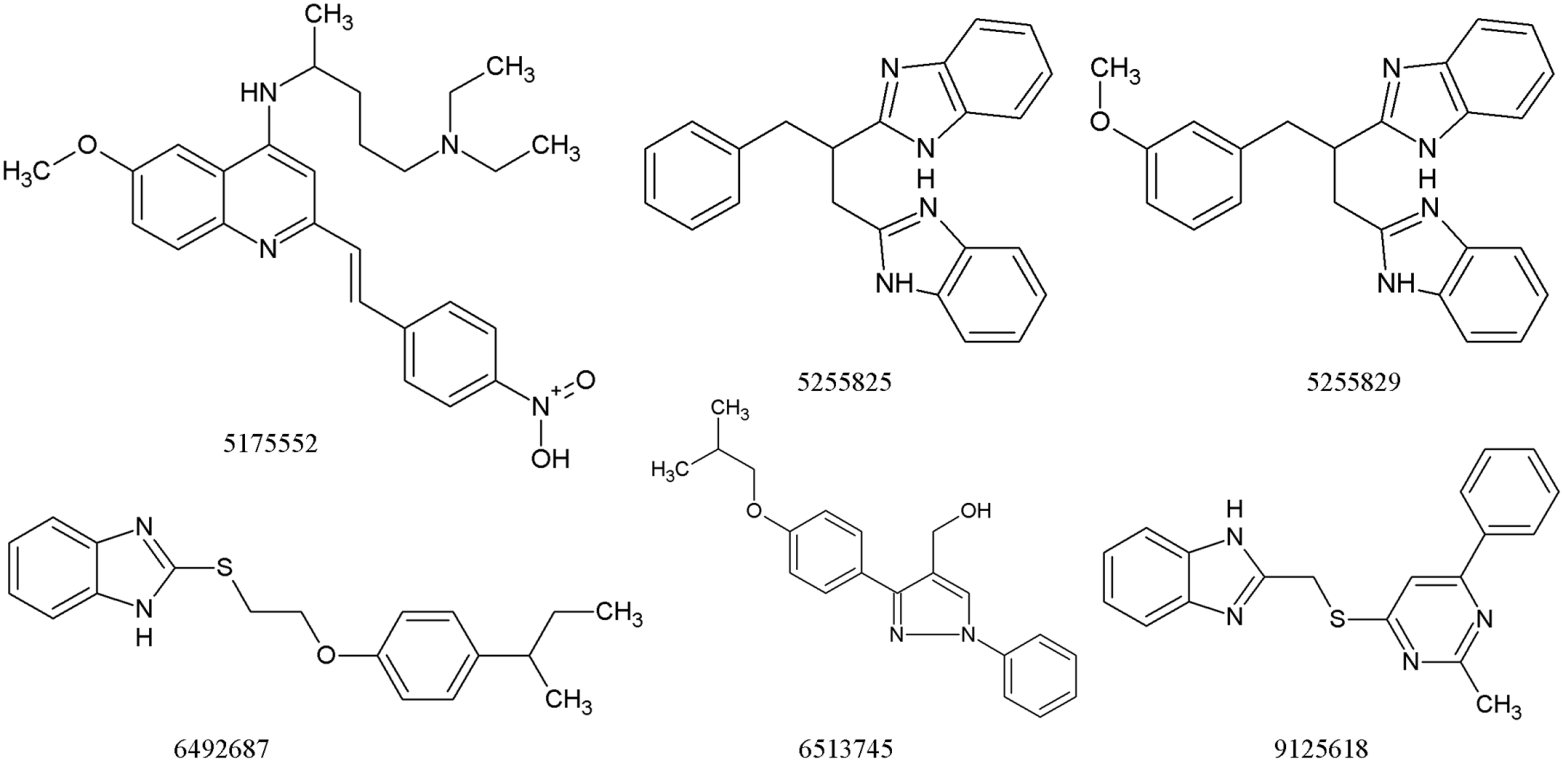
Structures of the top 6 compounds shortlisted by MelF flavin oxidoreductase activity and MIC/MBC determination.

The cytotoxic effect of top six compounds was examined in HeLa cells by MTT assay (Fig 11), which revealed that three compounds (# 5175552, # 5255829 and # 6513745) were not cytotoxic at both 1X and 5X MIC concentrations, whereas # 9125618 revealed ~ 18% cytotoxicity at 5X MIC concentration. On the other hand, two compounds (# 5255825, # 6492687) were cytotoxic at both the concentrations. It is pertinent to mention that out of three synergistic compounds identified for *M*. *tuberculosis* (Table 3), two showed cytotoxicity in HeLa cells; whereas one out of two synergistic compounds identified for *M*. *marinum* showed cytotoxicity in HeLa cells. Finally, compound # 5175552 and # 6513745, in combination with INH displayed synergistic inhibitory effect against *M*. *tuberculosis* and *M*. *marinum*, respectively with no cytotoxicity in HeLa cells. Interestingly, # 5175552 also displayed enhanced bactericidal effect (with MBC/MIC ratio of 1) in combination with RIF against *M*. *tuberculosis*. The chemical structures of six best compounds are shown in Fig 12 and their corresponding docked poses are depicted in (Fig 4A–4F). The docking scores and the basic molecular information about these inhibitors are given in S1 Table.

## Discussion

This study has led to the identification of relatively potent small molecule inhibitors for *M*. *marinum* MelF using VLS. A model for 3D structure of MelF protein was generated and its geometry-optimized surface scrutiny by multiple methods revealed a deep pocket with very high druggability index. Interestingly, a new and improved structure for Type II Baeyer-Villiger monooxygenase (5AEC) from *P*. *putida* has replaced the earlier 2WGK structure [19] after our VLS work was completed. However, a careful scrutiny reveals a very close similarity between the earlier 2WGK and the recently reported 5AEC structure (RMSD is ~ 0.15). In addition, a new structure of FMN bound enzyme (4UWM) has been submitted by the same group [19]. The occupancy of the cofactor modeled in the active site of newly submitted structure (4UWM) was ~ 0.6 thus indicating low occupancy of cofactor in the active site. This is also reflected in the poor electron density due to low affinity of FMN for Type II Baeyer-Villiger monooxygenase. A comparison of 4UWM and our docked compounds (Fig 4A–4F) also showed a partial overlap with the binding region of FMN (4UWM). In fact, the targeted druggable pocket is a juxtaposition of both cofactor (FMN) and the substrate binding sites. Notably, *in silico* analysis of the docked poses of our inhibitory compounds exhibited a dominance of van der Waals interaction, which has also been reported earlier [36,37] wherein the protein-compound interaction interface was deeply buried. However, an experimental validation by a crystal structure of MelF could have provided better explanation, but that is not determined till date.

VLS involves flexible-ligand docking into the pocket using the consensual approach among DOCK and Vina, which has also been successful in identifying ligands for several mycobacterial targets such as PknG (Rv1827), InhA (Rv1484), MtCM (Rv1885c), MurE (Rv2158c), DHQase (Rv2537c), L-AlaDH (Rv2780), UMPK (Rv2883c), EthR (Rv3855) [7,8,38–43] etc. In addition, the TB structural genomics consortium (TBSGC, http://www.webtb.org) determines the 3D structures of proteins from *M*. *tuberculosis* for rational structure-based drug designing [44]. The TBSGC is also actively developing bioinformatics resources that can be used for data mining to complement the structural information. Many of *M*. *tuberculosis* protein structures determined by TBSGC are associated with the metabolic pathways and are suggested as potential attractive anti-TB drug targets, which include structural studies on urease (Rv1848), chorismate-utilizing enzymes, arginine biosynthesis enzymes, crotonase, malate synthase (Rv1837c) and phosphoenolpyruvate carboxykinase (Rv0211), etc. [44–47]. Moreover, the atomic resolution studies of proteins from systems involving disulfide bond formation of the secreted proteins and toxin-antitoxin gene pairs can also be utilized for the structure-based drug design [44]. In this study, a series of compounds were predicted to dock into the active site and the shortlisted compounds met the Lipinski’s rule of 5 for drug-likeliness [26]. Of 178 putative inhibitors shortlisted, ~ 9% (16/178) were found to significantly inhibit the flavin oxidoreductase activity of purified MelF, whereas 1.68% (3/178) showed an MBC/MIC ratio of ≤ 2 for *M*. *marinum* and *M*. *tuberculosis* with no cytotoxic effect in HeLa cells. This further underlines the efficiency of VLS for the identification of novel protein activity modulators *vis-a-vis* chemical high throughput screening, whose success rate is ~ 0.01% [20].

As observed in bioluminescent bacteria, *M*. *marinum mel2* also facilitates the detoxification of ROS and RNS produced by the activated macrophages thus proposing that the bioluminescence systems might have been evolved from the oxidative defense mechanisms [11,12]. The *in silico* analysis of *mel2* locus has demonstrated a remarkable similarity to bioluminescent *lux* genes of *Vibrio harveyi* and the *melF* of *mel2* locus displays a high similarity to *luxA*, which has been shown to play a significant role in resistance to ROS in bioluminescent bacteria [12]. The presence of a *lux-*like operon, *mel2*, in non-bioluminescent pathogens of humans, that is, *M*. *tuberculosis* and *M*. *marinum* suggests that it could play a crucial role in mycobacterial pathogenesis [13,14], therefore, novel inhibitors against MelF flavin oxidoreductase activity were designed in the study and their bacteriostatic/bactericidal activity was examined against *M*. *marinum* and *M*. *tuberculosis in vitro*. Using *M*. *marinum* as an anti-TB activity evaluation model, Liu *et al*. [17] could identify an inhibitor targeting ICL (Rv0467) against both active and non-replicating *M*. *tuberculosis*. In fact, zebrafish *M*. *marinum* infection model has already been reported for investigating anti-TB agents *in vivo* as a high-throughput screening system [48].

Similar to mycobacterial MelF flavin oxidoreductase, *M*. *tuberculosis* dezaflavin-dependent nitroreducase (Ddn, Rv3547) and its two homologues Rv1261c and Rv1558 (which encode for an F_420_H_2_-dependent quinone reductase function, thus leading to the production of dihydroquinones) has been demonstrated to provide protection to mycobacterial cells from the oxidative stress and bactericidal agents [49]. Notably, the *M*. *tuberculosis* F_420_-deficient Ddn mutants defective in the formation of dezaflavin were found to be hypersensitive to INH, clofazimine and moxifloxacin thus proposing that the inhibitors that can inhibit Ddn or dezaflavin biosynthesis could synergize with the existing anti-TB drugs, as observed in this study also. These findings indicate the utility of exploiting MelF as a target for designing the inhibitors that might enhance the susceptibility of mycobacterial cells to oxidative stress thus leading to bacterial killing. We also speculate that the partial inhibition of MelF protein by inhibitors could possibly lead to accumulation of toxic intermediates thus resulting into bacterial killing.

Interestingly, Dwyer *et al*. [50] hypothesized that all bactericidal antibiotics kill bacteria by generating ROS/RNS, which has been demonstrated for the drugs such as delamanid, PA-824 and clofazimine for their activity against *M*. *tuberculosis* [15,16]. Perhaps, this explains the rational for the evolution of enzymes such as MelF flavin oxidoreductase and Ddn in imparting protection to mycobacterial cells against ROS/RNS. Further supporting the role of ROS in *M*. *tuberculosis* killing, Grant *et al*. [51] documented that even a small change in dissolved oxygen concentration (20%) could affect killing of mycobacterial persisters, a subpopulation of bacteria that is phenotypically resistant to killing by most of the antibiotics but still sensitive to high quantities of hydroxyl radicals. Notably, one inhibitor each for *M*. *marinum* and *M*. *tuberculosis* displayed synergistic inhibition in combination with INH, and two inhibitors displayed an enhanced bactericidal effect for *M*. *tuberculosis* in combination with RIF, with no cytotoxic effect in HeLa cells. The possible mechanism for such effects could be the triggering of ROS/RNS (produced by INH/RIF), which becomes more acute with the simultaneous blocking of MelF activity (with anti-ROS/RNS machinery) by the inhibitors. These findings suggest a need to further explore new drug-like molecules with the synergistic inhibitory effect and that may improve the efficacy of existing anti-TB drugs.

In addition to MelF flavin oxidoreductase and Ddn, other metabolic enzymes of *M*. *tuberculosis* have also been suggested as putative drug targets, which include lipoamide dehydrogenase (Lpd) and dihydrolipoamide acyltrasferase (DlaT), the components of pyruvate dehydrogenase [52] and *M*. *tuberculosis* peroxynitrite redutase/peroxidase function to resist RNS generated by the host cells [53]. By targeting the Lpd and DlaT, Bryk *et al*.[54,55] identified inhibitors that were selectively active against nonreplicating *M*. *tuberculosis*. Similarly, inhibitors of the respiratory chain and ATP synthesis have also been reported [56], which are probably the best-validated *M*. *tuberculosis* drug targets due to clinical success of the ATP synthase inhibitor TMC207 (bedaquiline) and the first-line TB drug pyrazinamide, which disrupts the proton motive force [57].

We anticipate that the inhibitors inhibiting the flavin oxidoreductase activity of *M*. *marinum* MelF would also inhibit *M*. *tuberculosis* MelF activity. Several researchers have targeted similar enzyme systems of *E*. *coli* and *M*. *tuberculosis*. For example, drugs such as sulfonylureas, imidazolinones and benzoyl esters, the traditional inhibitors of *E*. *coli* acetohydroxyacid synthases (AHAS) also inhibit *M*. *tuberculosis* AHAS [58]. Furthermore, the use of *M*. *tuberculosis* 3-isopropylmalate dehydrogenase (IPMDH) has been exploited as a drug target [59], which has homologues in other bacterial species such as *E*. *coli* and *Thermus thermophilus* and the inhibitory effect of IPMDH inhibitors was found to be independent of the origin of IPMDH. Similarly, the inhibitors targeting *M*. *marinum* MelF in the study also inhibited *M*. *tuberculosis in vitro* independent of the origin of MelF.

In conclusion, we cloned, expressed and purified *M*. *marinum* MelF protein. This is the first report that establishes the flavin dependent oxidoreductase activity of *M*. *marinum* MelF. The potential inhibitors targeting MelF were identified through *in silico* analysis, which inhibited the flavin oxidoreductase activity of MelF and showed bacteriostatic/bactericidal activity against *M*. *marinum* and *M*. *tuberculosis in vitro*. Interestingly, two inhibitors also displayed synergistic inhibitory effect in combination with INH against *M*. *marinum*/*M*. *tuberculosi*s with no cytotoxic effect in HeLa cells. Further work is in progress to examine the effect of these inhibitors in human and mouse activated macrophages infected with *M*. *marinum* and *M*. *tuberculosis*.

## Supporting information

S1 Table Properties of top six compounds shortlisted based on MelF flavin oxidoreductase activity and MIC/MBC values. (DOCX)

S2 Table α-helical and β-sheets content of MelF by CD spectra in presence and absence inhibitors. (DOCX)

S3 Table WHATIF results. (DOCX)

S1 Fig Cloning of *M*. *marinum melF*: Confirmation of cloned melF via fragment release by BamH1 and HindIII. (TIF)

S2 Fig Stereo image of the targeted site. The site as shown in grey wire mesh was selected as consenual site, as predicted by InCaSiteFinder, Q-SiteFinder and PocketFinder.
(TIF)

S3 Fig Sequence alignment between MelF of *M*. *marinum* and *M*. *tuberculosis* H_37_Rv showing 98% sequence identity. (TIF)

S4 Fig Ramachandran plot for 3D structure of MelF. (TIF)

S5 Fig Graph between the docking scores (Autodock-Vina) and K_i_ values. (TIF)
