# Supplementary material for: Rational design of drug-like compounds targeting Mycobacterium marinum MelF protein
Source: PLoS One. 2017 Sep 5;12(9):e0183060. doi: 10.1371/journal.pone.0183060 (PMC5584760; doi:10.1371/journal.pone.0183060)
Supplement: S1 Table — (DOCX) [file pone.0183060.s001.docx]

**S1 Table**: Properties of top six compounds shortlisted based on MelF flavin oxidoreductase activity and MIC/MBC values

| **Molecule Properties** | **5175552** | **5255825** | **5255829** | **6492687** | **6513745** | **9125618** |
| --- | --- | --- | --- | --- | --- | --- |
| **IUPAC Name** | [3-(4-isobutoxyphenyl)-1-phenyl-1H-pyrazol-4-yl]methanol | 2,2'-(3-phenyl-1,2-propanediyl)bis-1H-benzimidazole | N~1~,N~1~-diethyl-N~4~-{6-methoxy-2-[2-(4-nitrophenyl)vinyl]-4-quinolinyl}-1,4-pentanediamine | 2-{[2-(4-sec-butylphenoxy)ethyl]thio}-1H-benzimidazole | 2-{[(2-methyl-6-phenyl-4-pyrimidinyl)thio]methyl}-1H-benzimidazole | 2,2'-[3-(3-methoxyphenyl)-1,2-propanediyl]bis-1H-benzimidazole |
| **Mass** | 463 | 322 | 382 | 326 | 352 | 332 |
| **H-Bond Donor** | 2 | 1 | 2 | 1 | 2 | 1 |
| **H-Bond Acceptor** | 5 | 3 | 3 | 2 | 2 | 3 |
| **LogP Value** | 4.82 | 4.25 | 5.01 | 5.24 | 5.008 | 4.43 |
| **Molar Refractivity** | 139.54 | 95.41 | 115.3 | 97.40 | 108.77 | 97.182 |
| **Estimated Binding Energy** | -11.369 | -10.126 | -10.121 | -8.874 | -8.389 | -8.227 |
| **Ki Values** | 68 | 4 | 9 | 25 | 18 | 24 |
| **Binding Residues** | F7, L8, M9, V42, G43, E44, H45, E51, P74, G75, A76, H77, L78, G110, T185, G186, F187, S188, S191, L207, D208, L209, V204, R241, E242, V269, T302, F303, L331 | F7, M9, H45, E51, F187, L207, D208, L209, V240, R241, E242, M265, V269, Y282, F281, P286 L298, N301, T302, F303, L331 | F7, M9, H45, W50, E51, F187, L207, D208, L209, V240, E242, M265, V269, Y282, L298, T302, L331 | F7, M9, V42, H45, E51, A76, S109, T185, F187, L207, D208, L209, V240, E242, M265, V269, F281, Y282, P286, L298 N301, T302, F303, L331 | F7, M9, W50, E51, F187, M205, L207, D208, L209, V240, E242, M265, Y268, V269, Y282, L298, T302, F303, L331 | F7, M9, V42 G43, E44, H45, E51, G75, A76, H77, L78, S109, G110, I111, T185, F187, S188, L207, V240, E242 |
