# Supplementary material for: Rational design of drug-like compounds targeting Mycobacterium marinum MelF protein
Source: PLoS One. 2017 Sep 5;12(9):e0183060. doi: 10.1371/journal.pone.0183060 (PMC5584760; doi:10.1371/journal.pone.0183060)
Supplement: S2 Table — (DOCX) [file pone.0183060.s002.docx]

|  | **MelF** | **MelF+5175552** | **MelF+5255825** | **MelF+5255829** | **MelF+6492687** | **MelF+6513745** | **MeiF+9125618** |
| --- | --- | --- | --- | --- | --- | --- | --- |
| α-Helices | 91.77 | 91.76 | 93.21 | 91.76 | 94.75 | 93.72 | 91.77 |
| β-Sheets | 0.01 | 0.01 | 0.01 | 0.01 | 0.01 | 0.01 | 0.01 |

S2 Table: α-helical and β-sheets content of MelF by CD spectra in presence and absence inhibitors
