# Supplementary material for: Rational design of drug-like compounds targeting Mycobacterium marinum MelF protein
Source: PLoS One. 2017 Sep 5;12(9):e0183060. doi: 10.1371/journal.pone.0183060 (PMC5584760; doi:10.1371/journal.pone.0183060)
Supplement: S3 Table — (DOCX) [file pone.0183060.s003.docx]

**WHATIF results**

# Name check.

Torsion angles that need flipping for proper nomenclature. The residues are sorted by residue type.

Date= 2017-01-06 09:56:01

No errors were detected in valine nomenclature.

No errors were detected in threonine nomenclature.

No errors were detected in isoleucine nomenclature.

No errors were detected in leucine nomenclature.

The arginine residues listed in the table below have their N-H-1 and N-H-2

swapped.

16 ARG ( 16 ) A

66 ARG ( 66 ) A

90 ARG ( 90 ) A

The tyrosine residues listed in the table below have their chi-2 not between

-90.0 and 90.0

37 TYR ( 37 ) A

81 TYR ( 81 ) A

154 TYR ( 154 ) A

The phenylalanine residues listed in the table below have their chi-2 not

between -90.0 and 90.0.

7 PHE ( 7 ) A

46 PHE ( 46 ) A

102 PHE ( 102 ) A

187 PHE ( 187 ) A

303 PHE ( 303 ) A

The aspartic acid residues listed in the table below have their chi-2 not

between -90.0 and 90.0, or their proton on OD1 instead of OD2.

25 ASP ( 25 ) A

57 ASP ( 57 ) A

219 ASP ( 219 ) A

334 ASP ( 334 ) A

The glutamic acid residues listed in the table below have their chi-3

outside the -90.0 to 90.0 range, or their proton on OE1 instead of OE2.

15 GLU ( 15 ) A

30 GLU ( 30 ) A

39 GLU ( 39 ) A

212 GLU ( 212 ) A

223 GLU ( 223 ) A

351 GLU ( 351 ) A

352 GLU ( 352 ) A

No errors were detected in the atom names for non-hydrogen atoms.

# Coarse Packing Quality Control

The packing quality control per amino acid:

Date= 2017-01-06 10:01:05

Average for range 1 - 366 : -1.606

If a residue has a score of -5.0 or lower, something is really going on: the residue makes symmetry contacts, is contacting a ligand or an ion, or something is wrong.

**Planarity validation results:**

The side chains of the residues listed in the table below contain a planar

group that was found to deviate from planarity by more than 4.0 times the

expected value. For an amino acid residue that has a side chain with a

planar group, the RMS deviation of the atoms to a least squares plane was

determined. The number in the table is the number of standard deviations

this RMS value deviates from the expected value. Not knowing better yet, we

assume that planarity of the groups analyzed should be perfect.

12 HIS ( 12 ) A 5.67

96 HIS ( 96 ) A 5.03

290 ASP ( 290 ) A 4.94

255 TYR ( 255 ) A 4.31

273 PHE ( 273 ) A 4.22

The atoms listed in the table below are connected to a planar aromatic group

in the sidechain of a protein residue but were found to deviate from the

least squares plane.

For all atoms that are connected to an aromatic side chain in a protein

residue the distance of the atom to the least squares plane through the

aromatic system was determined. This value was divided by the standard

deviation from a distribution of similar values from a database of small

molecule structures.

142 TRP ( 142 ) A CB 5.35

335 TYR ( 335 ) A OH 5.24

118 TYR ( 118 ) A OH 5.02

37 TYR ( 37 ) A OH 4.37

Since there is no DNA and no protein with hydrogens, no uncalibrated

planarity check was performed.

# Anomalous bond lengths.

Bond lengths that deviate more than 4 sigma:

Date= 2017-01-06 10:35:42

The bond lengths listed in the table below were found to deviate more than 4

sigma from standard bond lengths (both standard values and sigmas for amino

acid residues have been taken from Engh and Huber [REF], for DNA they were

taken from Parkinson et al [REF]). In the table below for each unusual bond

the bond length and the number of standard deviations it differs from the

normal value is given.

Atom names starting with "-" belong to the previous residue in the chain. If

the second atom name is "-SG*", the disulphide bridge has a deviating length.

10 PRO ( 11 ) A N CA 1.39 -4.8

Bond lengths were found to deviate less than normal from the mean Engh and

Huber [REF] and/or Parkinson et al [REF] standard bond lengths. The RMS

Z-score given below is expected to be near 1.0 for a normally restrained

data set. The fact that it is lower than 0.667 in this structure might

indicate that too-strong restraints have been used in the refinement. This

can only be a problem for high resolution X-ray structures.

RMS Z-score for bond lengths: 0.520

RMS-deviation in bond distances: 0.011

Comparison of bond distances with Engh and Huber [REF] standard values for

protein residues and Parkinson et al [REF] values for DNA/RNA shows a

significant systematic deviation. It could be that the unit cell used in

refinement was not accurate enough. The deformation matrix given below gives

the deviations found: the three numbers on the diagonal represent the

relative corrections needed along the A, B and C cell axis. These values are

1.000 in a normal case, but have significant deviations here (significant at

the 99.99 percent confidence level)

There are a number of different possible causes for the discrepancy. First

the cell used in refinement can be different from the best cell calculated.

Second, the value of the wavelength used for a synchrotron data set can be

miscalibrated. Finally, the discrepancy can be caused by a dataset that has

not been corrected for significant anisotropic thermal motion.

Please note that the proposed scale matrix has NOT been restrained to obey

the space group symmetry. This is done on purpose. The distortions can give

you an indication of the accuracy of the determination.

If you intend to use the result of this check to change the cell dimension

of your crystal, please read the extensive literature on this topic first.

This check depends on the wavelength, the cell dimensions, and on the

standard bond lengths and bond angles used by your refinement software.

0.018194 0.000000 0.000000

0.000000 0.010716 0.000000

0.000000 0.000000 0.006177

0.998361 -0.000065 0.000120

-0.000065 0.998038 -0.000166

0.000120 -0.000166 0.999321

0.018224 0.000001 -0.000002

0.000000 0.010737 0.000002

0.000000 0.000001 0.006181

Variance: 53.380

(Under-)estimated Z-score: 5.385

# Highly likely peptide flips

Peptide flip validation results:

Date= 2017-01-06 10:36:41

There was no need to complain about the peptide bond of a single amino acid

# Relaxed peptide flips

Relaxed peptide flip validation results:

Date= 2017-01-06 10:37:14

For the residues listed in the table below, the backbone formed by the

residue mentioned and the one N-terminal of it show systematic deviations

from normality that are consistent with a peptide flip. This can either

be a 180 degree flip of the entire peptide plane or a trans to cis flip.

(Cis to trans flips cannot be detected yet). The type can be TT+, TC-,

or TC+:

TT+ indicates a 180 degree flip of the entire peptide plane.

TC- indicates a trans to cis conversion that requires a flip of the N atom.

TC+ indicates a trans to cis conversion that requires a flip of the O atom.

Note that the method will only work correctly for PDB files with full

isotropic B-factors.

194 ASN ( 206 ) A TT+ Somewhat likely

486 ASN ( 123 ) B TT+ Somewhat likely

# Fine Packing Quality Control

The packing quality control per amino acid:

Date= 2017-01-06 10:37:48

----Residue------- State AllAll BB-BB BB-SC SC-BB SC-SC

---------------------------------------------------------------------------

All contacts : Average = -0.369 Z-score = -2.42

BB-BB contacts : Average = -0.235 Z-score = -1.61

BB-SC contacts : Average = -0.255 Z-score = -2.01

SC-BB contacts : Average = -0.347 Z-score = -2.01

SC-SC contacts : Average = -0.342 Z-score = -1.83

If an individual residue has a quality control value of -2.5 or worse, you should take a look at it. It can mean that the residue:

- is involved in symmetry contacts, or
- is binding a co-factor, ligand or ion, or
- is an active site residue, or
- is wrong.

Average protein values ("Z-score for all contacts") can be read as follows:

- -5.0 Guaranteed wrong structure. Bad structure or poor model
- -3.0 Probably bad structure or unrefined model. Doubtful structure or model
- -2.0 Structure OK or good model. Good structures
- 0.0 Good structures.
- 2.0 Good structures. Unusually Good structures
- 4.0 Probably a strange model of a perfect helix

# Collisions with symmetry axes.

Atoms that are too close to a symmetry axis:

Date= 2017-01-06 10:38:59

Either there were no atoms at special positions, or all atoms at special

positions have adequately reduced occupancies. An atom is considered to be

located at a special position if it is within 0.3 Angstrom from one of its

own symmetry copies. See also the next check...

None of the atoms in the structure is closer than 0.77 Angstrom to a proper

symmetry axis.

# Hand check.

Atoms with a chirality that is more than4 sigma away from normal:

Date= 2017-01-06 10:41:00

No atoms are observed that have the wrong handedness. Be aware, though, that

WHAT CHECK might have corrected the handedness of some atoms already. The

handedness has not been corrected for any case where the problem is worse

than just an administrative discomfort.

All protein atoms have proper chirality, or there is no intact protein

present in the PDB file.

The average deviation= 1.003

The RMS Z-score for all improper dihedrals in the structure is within normal

ranges.

Improper dihedral RMS Z-score : 0.831

# Omega.

Omega validation results:

Date= 2017-01-06 10:42:18

The omega angles for trans-peptide bonds in a structure is expected to give a

gaussian distribution with the average around +178 degrees, and a standard

deviation around 5.5. In the current structure the standard deviation agrees

with this expectation.

Omega average and std. deviation= 179.230 5.825

# Proline puckering.

Prolines with funny puckers:

Date= 2017-01-06 10:43:20

The proline residues listed in the table below have a puckering amplitude

that is outside of normal ranges. Puckering parameters were calculated by

the method of Cremer and Pople [REF]. Normal PRO rings have a puckering

amplitude Q between 0.20 and 0.45 Angstrom. If Q is lower than 0.20 Angstrom

for a PRO residue, this could indicate disorder between the two different

normal ring forms (with C-gamma below and above the ring, respectively). If

Q is higher than 0.45 Angstrom something could have gone wrong during the

refinement. Be aware that this is a warning with a low confidence level. See:

Who checks the checkers? Four validation tools applied to eight atomic

resolution structures [REF]

49 PRO ( 49 ) A 0.60 HIGH

56 PRO ( 56 ) A 0.20 LOW

181 PRO ( 181 ) A 0.51 HIGH

The proline residues listed in the table below have a puckering phase that is

not expected to occur in protein structures. Puckering parameters were

calculated by the method of Cremer and Pople [REF]. Normal PRO rings

approximately show a so-called envelope conformation with the C-gamma atom

above the plane of the ring (phi=+72 degrees), or a half-chair conformation

with C-gamma below and C-beta above the plane of the ring (phi=-90 degrees).

If phi deviates strongly from these values, this is indicative of a very

strange conformation for a PRO residue, and definitely requires a manual

check of the data. Be aware that this is a warning with a low confidence

level. See: Who checks the checkers? Four validation tools applied to eight

atomic resolution structures [REF].

147 PRO ( 147 ) A -62.8 half-chair C-beta/C-alpha (-54 degrees)

174 PRO ( 174 ) A -40.6 envelop C-alpha (-36 degrees)

204 PRO ( 204 ) A -64.0 envelop C-beta (-72 degrees)

271 PRO ( 271 ) A 99.2 envelop C-beta (108 degrees)

295 PRO ( 295 ) A 101.9 envelop C-beta (108 degrees)

341 PRO ( 341 ) A -61.0 half-chair C-beta/C-alpha (-54 degrees)

# Anomalous bond angles.

Bond angles that deviate more than 4 sigma:

Date= 2017-01-06 10:43:50

The bond angles listed in the table below were found to deviate more than 4

sigma from standard bond angles (both standard values and sigma for protein

residues have been taken from Engh and Huber [REF], for DNA/RNA from

Parkinson et al [REF]). In the table below for each strange angle the bond

angle and the number of standard deviations it differs from the standard

values is given. Please note that disulphide bridges are neglected. Atoms

starting with "-" belong to the previous residue in the sequence.

Bond angles were found to deviate normally from the mean standard bond angles

(normal values for protein residues were taken from Engh and Huber [REF], for

DNA/RNA from Parkinson et al [REF]). The RMS Z-score given below is expected

to be near 1.0 for a normally restrained data set, and this is indeed

observed for very high resolution X-ray structures.

RMS Z-score for bond angles: 0.753

RMS-deviation in bond angles: 1.509

# Checking water and ion switches.

Information about the status of ions and waters

Date= 2017-01-06 10:46:11

Since there are no ions in the structure of a type we can validate, this

check will not be executed.

Since there are no waters, the water check has been skipped.

**
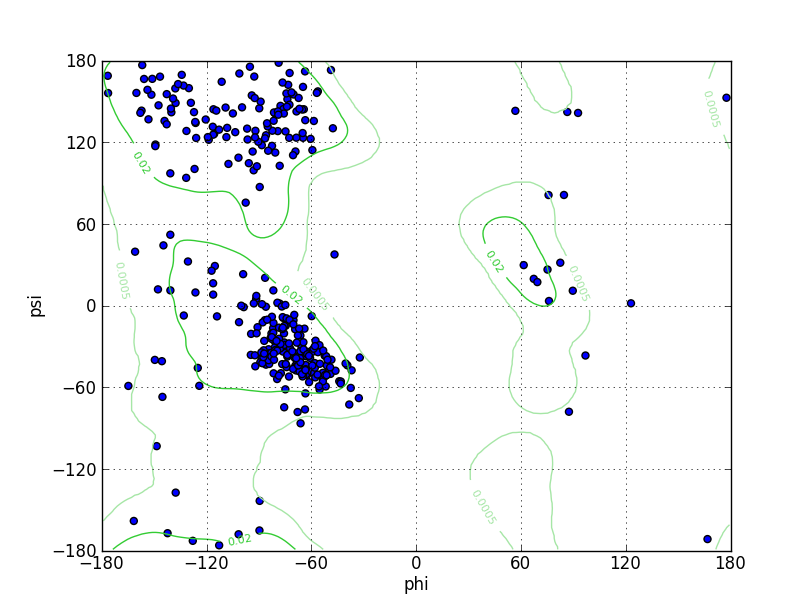
Ramachandran PLOT**
