# Supplementary figures and images for: Rational design of drug-like compounds targeting Mycobacterium marinum MelF protein
Source: PLoS One. 2017 Sep 5;12(9):e0183060. doi: 10.1371/journal.pone.0183060 (PMC5584760; doi:10.1371/journal.pone.0183060)

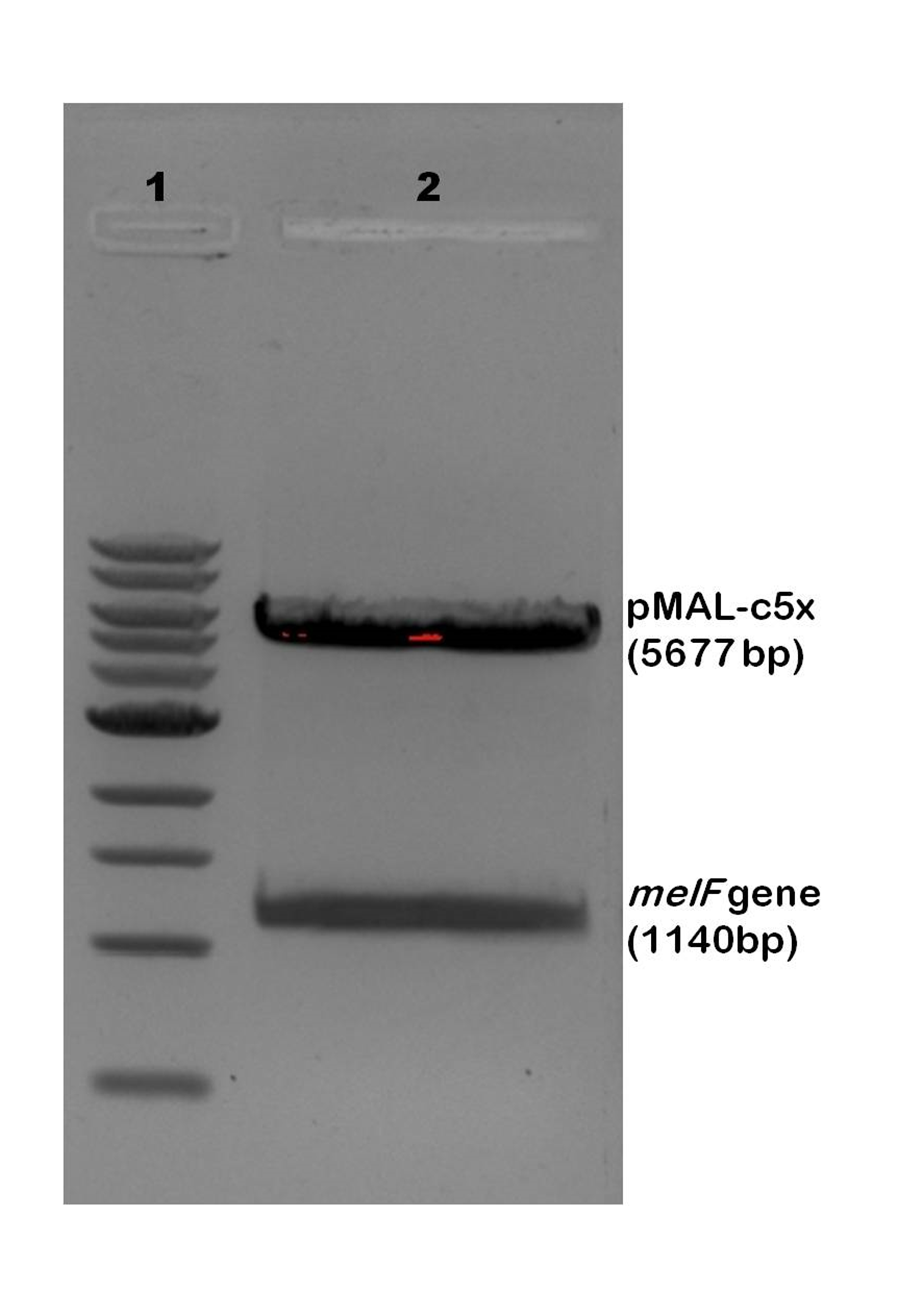

Supplement: S1 Fig — (TIF) [file pone.0183060.s004.tif]

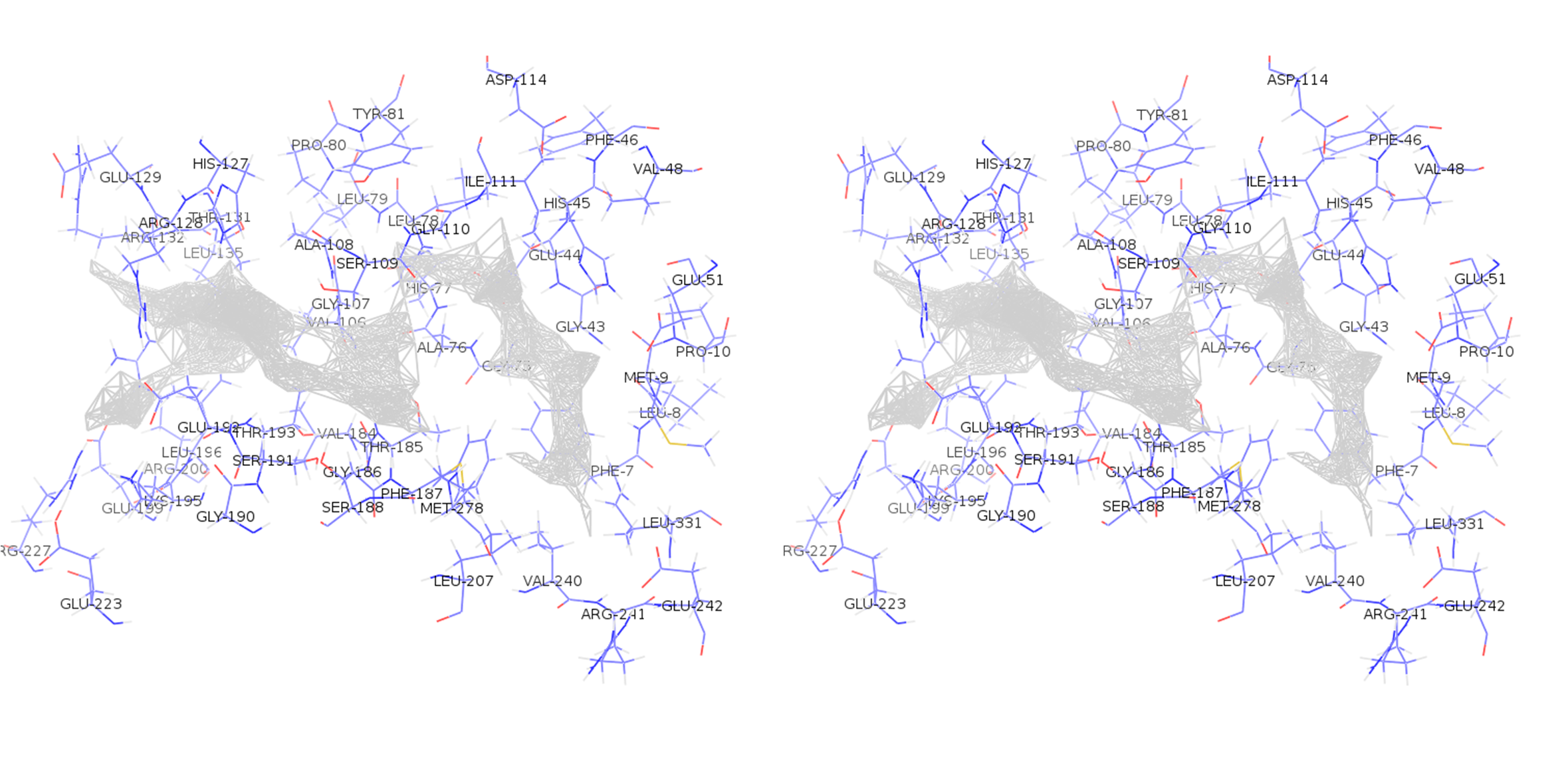

Supplement: S2 Fig — The site as shown in grey wire mesh was selected as consenual site, as predicted by InCaSiteFinder, Q-SiteFinder and PocketFinder. (TIF) [file pone.0183060.s005.tif]

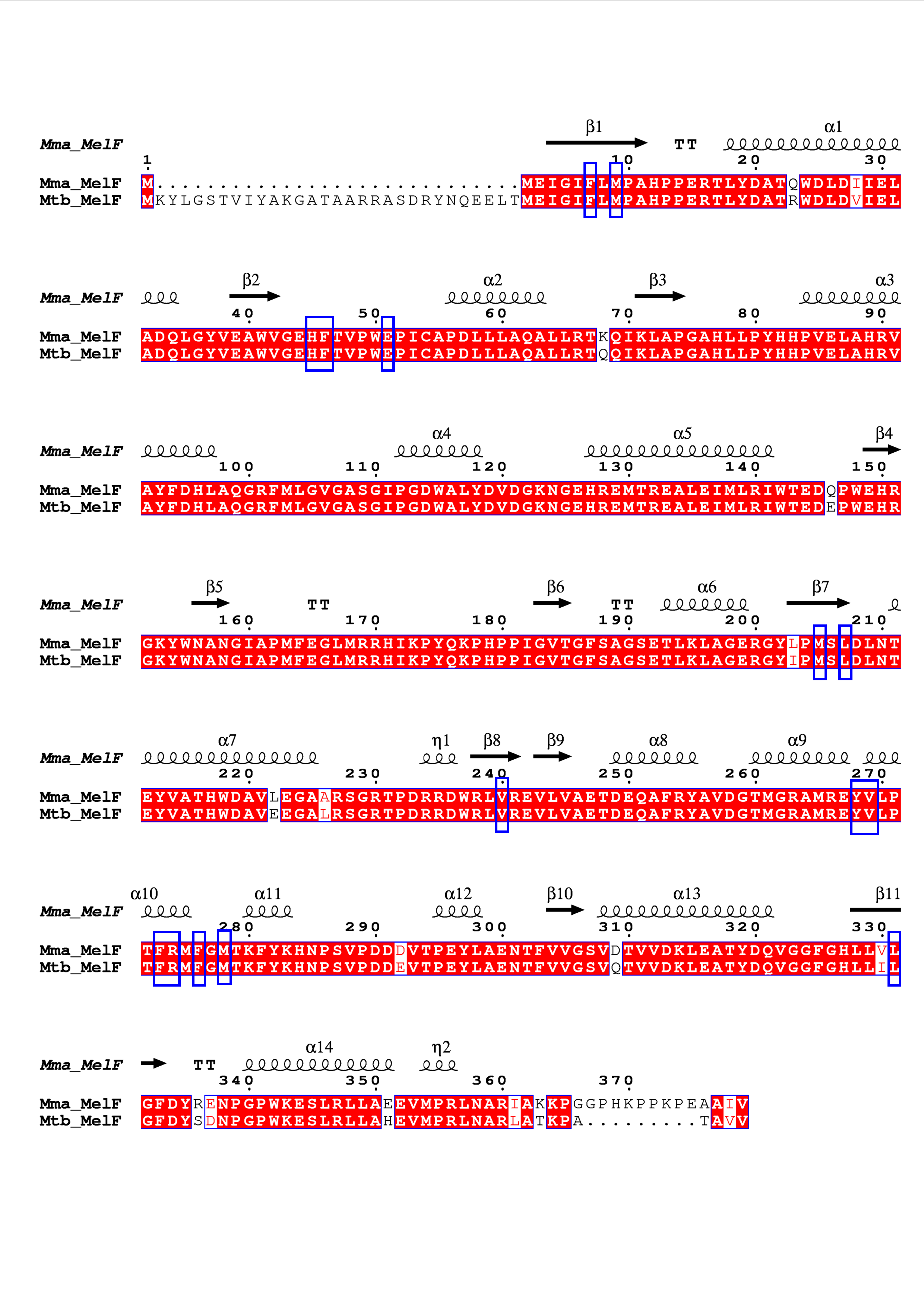

Supplement: S3 Fig — (TIF) [file pone.0183060.s006.tif]

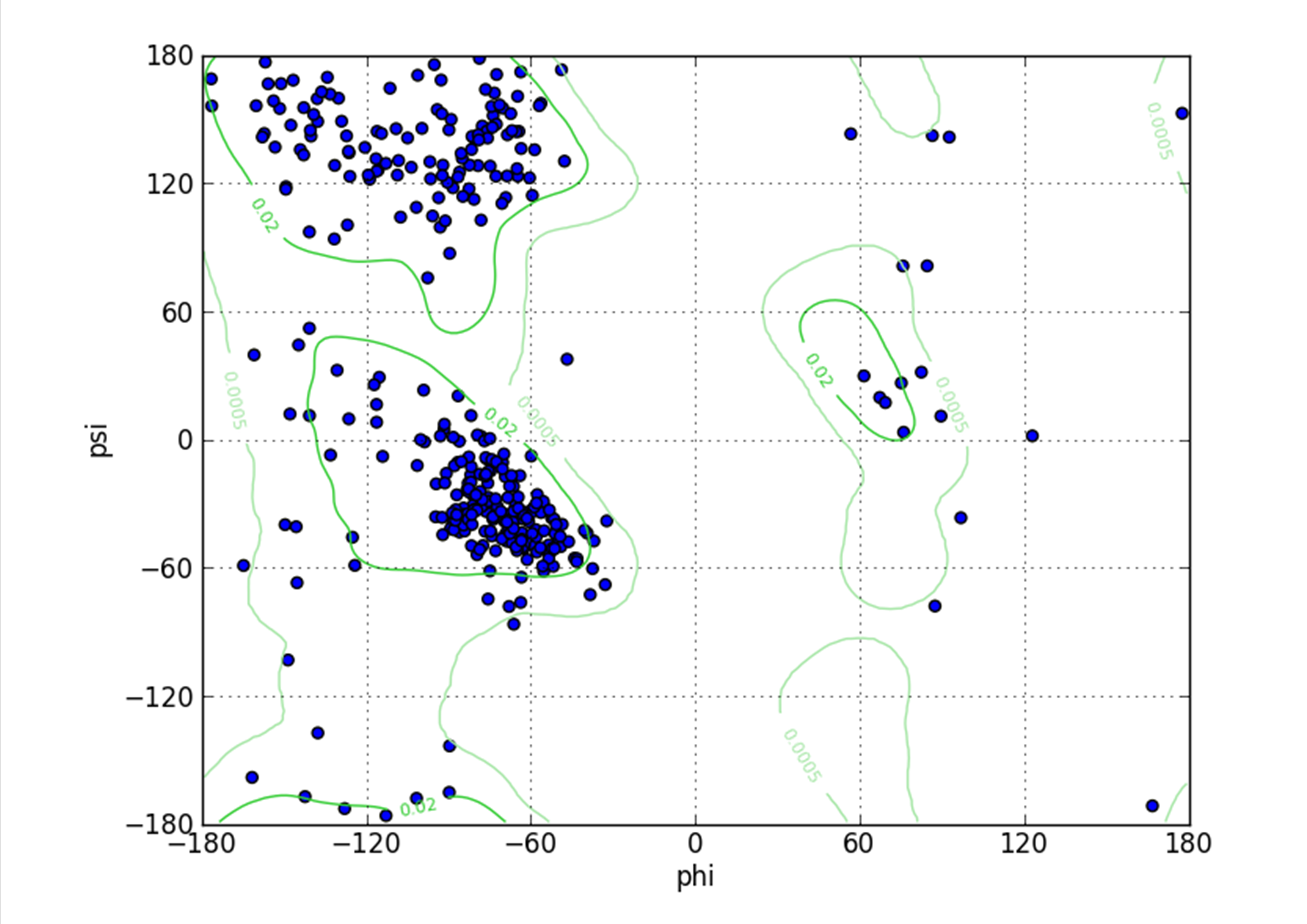

Supplement: S4 Fig — (TIF) [file pone.0183060.s007.tif]

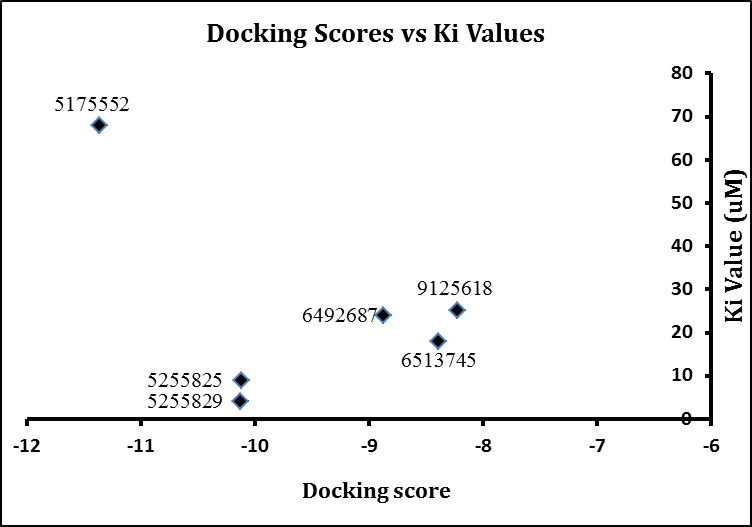

Supplement: S5 Fig — (TIF) [file pone.0183060.s008.tif]
